# Supplementary material for: Comparison of biosimilar Tigerase and Pulmozyme in long-term symptomatic therapy of patients with cystic fibrosis and severe pulmonary impairment (subgroup analysis of a Phase III randomized open-label clinical trial (NCT04468100))
Source: PLoS One. 2021 Dec 23;16(12):e0261410. doi: 10.1371/journal.pone.0261410 (PMC8699637; doi:10.1371/journal.pone.0261410)
Supplement: S1 File — (PDF) [file pone.0261410.s003.pdf]

CLINICAL STUDY PROTOCOL

**Protocol No.:**	DRN-CFR-II/III
**Version:**	1.0
**Date:**	April 4, 2017
**Study title:**	An open-label, prospective, multicenter, randomized comparative study of pharmacokinetics, clinical effectiveness, and safety of Tigerase (GENERIUM JSC, Russia) and Pulmozyme® (F. Hoffmann-La Roche Ltd., Switzerland) as a part of the combined therapy in the patients with cystic fibrosis
**Study phase:**	II/III
**Sponsor:**	Generium JSC, Russia<br>Registered office: Bldg. 17, Volginsky settlement, Petushinsky District,<br>Vladimir Region, 601125 Russia<br><br>Moscow office: 10 Testovskaya Street, Entrance 2, Moscow, 123112 Russia;<br>Phone/Fax: +7 (495) 988-47-94<br>generium@generium.ru / www.generium.ru

Protocol version:
This version 1.0 dated April 4, 2017, is an initial document.

GENERAL INFORMATION ON CLINICAL STUDY AND AUTHORIZED SIGNATURE

**Study title:**	An open-label, prospective, multicenter, randomized comparative study of pharmacokinetics, clinical effectiveness, and safety of Tigerase (GENERIUM JSC, Russia) and Pulmozyme® (F. Hoffmann-La Roche Ltd., Switzerland) as a part of the combined therapy in the patients with cystic fibrosis	
**Study protocol:**	DRN-CFR-II/III, version 1.0	April 4, 2017
**Study sponsor:**	GENERIUM JSC,<br>[redacted].<br>Moscow office:<br>[redacted]	
**General Director, GENERIUM JSC**	[redacted]	____________________<br>*signature*<br>____________________<br>*date*
**Clinical study site:**	The study will be conducted at the clinical centers accredited by the Ministry of Health of the Russian Federation to carry out the clinical studies of the medicinal products for human use.	

PAGE OF PROTOCOL APPROVAL BY INVESTIGATOR

An open-label, prospective, multicenter, randomized comparative study of pharmacokinetics, clinical effectiveness, and safety of Tigerase (GENERIUM JSC, Russia) and Pulmozyme® (F. Hoffmann-La Roche Ltd., Switzerland) as a part of the combined therapy in the patients with cystic fibrosis		
Protocol	DRN-CFR-II/III	Version 1.0 dated April 4, 2017

I, the undersigned, am responsible for conducting the study at this site and agree with the following:

- I hereby confirm that I have read the latest revision of this protocol, including all appendices hereto, and the Instruction for Use of the Investigational Product and understood the information specified in these documents.
- I confirm that I have enough time for correct performance and completion of the study within its stipulated period, and I have enough qualified employees and material resources (including premises and equipment) for the adequate and safe performance of this study.
- I undertake to carry out the clinical study in accordance with the conditions, terms, and procedures of this protocol in compliance with the national and international principles of ICH GCP (Good Clinical Practice) and all regulatory requirements of the Russian Federation applicable legislation in the field of the medicines circulation (including National Standard of the Russian Federation GOST R 52379-2005, Good Clinical Practice, Order of the Ministry of Health of the Russian Federation No. 200n (200н) dated April 1, 2016, “Concerning Approval of the Rules for Good Clinical Practice”).
- I undertake to ensure that all employees involved in the clinical study are familiar with the protocol and the investigational drug Tigerase (GENERIUM JSC, Russia) and the reference product Pulmozyme® (F. Hoffmann-La Roche Ltd., Switzerland), as well as with their functions and responsibilities.
- I also give my consent to the following:
  – compliance with the procedure of the registration and submission of the clinical trial data;
  – monitoring and auditing of this clinical study;
  – storage of the clinical trial documents until the latter be allowed to be destroyed by the sponsor.

**Full name, position of Principal Investigator**	**Signature**	**Date**
		____________

CONTACT INFORMATION

Medical Monitor,<br>GENERIUM JSC	[redacted]
Analytical laboratory	[redacted]
Provision of Product Safety Reports	GENERIUM JSC<br>e-mail: [redacted]<br>Fax: [redacted]

TABLE OF CONTENTS

GENERAL INFORMATION ON CLINICAL STUDY AND AUTHORIZED SIGNATURE 3
PAGE OF PROTOCOL APPROVAL BY INVESTIGATOR 4
CONTACT INFORMATION 5
TABLE OF CONTENTS 6
ABBREVIATIONS 12
PROTOCOL ABSTRACT 14
1 STUDY RATIONALE 25
1.1 Introduction 25
1.2 Name and Description of the Investigational Product 29
1.2.1 Physical and Chemical Properties of Active Substance Dornase Alfa 29
1.3 Preclinical and Clinical Study Results 29
1.3.1 Preclinical Safety Data 29
1.3.1.1 Pharmacokinetics and Metabolism in Animals 29
1.3.1.2 Toxicity Studies 31
1.3.2 Findings of Phase I Clinical Study of Tigerase 33
1.3.3 Clinical Study of Original Medicinal Product Pulmozyme® 35
1.4 Risk–Benefit Ratio Assessment for Study Subjects 41
1.4.1 Risks Associated with the Use of the Investigational Product 41
1.4.1.1 Warnings and Precautions for Use 41
1.4.1.2 Overdose 42
1.4.1.3 Risks Associated with the Use of the Study/Reference Product 42
1.4.2. Risks Associated with the Study Diagnostic Procedures 42
1.5 Rationale for Reference Product, Administration, and Dosage Schedule 43
1.6 Study Quality Statement 44
1.7 Study Population Description 44
1.8 References 45
2 STUDY PURPOSE AND OBJECTIVES 51
3 STUDY PROCEDURE 52

3.1 Study Design 52
3.2 Study Methodology 52
3.2.1 Measures to Minimize/Exclude Subjectivity in Clinical Study 53
3.2.1.1 Randomization Procedure..53
3.2.1.2 Blinding Procedure 54
3.3 Graphical Flowchart of the Study 54
4 STUDY POPULATION 56
4.1 Subject Inclusion Criteria 56
4.2 Subject Exclusion Criteria 56
4.3 Premature Withdrawal from Study and Subject Replacement Procedure 57
4.3.1 Withdrawal Criteria 57
4.4 Data Collection and Follow-Up of the Early Withdrawing Subjects 58
4.4.1 Replacement of Withdrawn Subjects 58
5 INFORMATION ON THE STUDY/REFERENCE PRODUCT 59
5.1 Pharmaceutical Dosage Form, Composition, and Clinical Pharmacology of the Investigational Product..59
5.2 Pharmaceutical Dosage Form, Composition, and Clinical Pharmacology of the Reference Product 59
5.3 Storage Conditions of the Study/Reference Product 60
5.4 Accountability of the Study/Reference Product 60
5.5 Dosing Schedule of the Study/Reference Product 61
5.6 Preparation of the Study/Reference Product for Use 61
6 PROTOCOL COMPLIANCE 63
6.1 Measures to Ensure Protocol Compliance..63
6.2 Patient Compliance...63
7 CONCOMITANT THERAPY, PROHIBITED MEDICATIONS AND THERAPY METHODS 63
7.1 Concomitant Therapy..63
7.2 Prohibited Medicinal Products 63
7.3 Prohibited Therapy Methods...63
8 EXAMINATION PLAN AND VISITS 64

8.1 Patient Enrollment 64
8.2 Visit 1. Screening/Enrollment to Study (Days -21 to -1) 64
9 TREATMENT PERIOD 66
9.1 Visit 2. Day 1. Randomization (Week 0) 66
9.2 Visit 3. Day 7 ± 2 days (Week 1) 67
9.3 Visit 4. Telephone Call. Day 28 ± 3 days (Week 4) 67
9.4 Visit 5. Day 56 ± 7 days (Week 8) 67
9.5 Visit 6. Telephone Call. Day 84 ± 7 days (Week 12) 68
9.6 Visit 7. Day 112 ± 7 days (Week 16) 68
9.7 Visit 8. Telephone Call. Day 140 ± 7 days (Week 20) 68
9.8 Visit 9. Day 168 ± 7 days (Week 24 ± 1 week) (End of Therapy Visit / Early Termination Visit, EOT/ET) 68
9.9 Unscheduled Visits 69
10 STUDY PROCEDURES 69
10.1 Collection of Demographics and Medical History 69
10.2 Anthropometry 70
10.3 Physical Examination 70
10.4 Vital Signs 71
10.5 Clinical Symptoms of Chronic Pulmonary Disease Exacerbation 71
10.6 Telephone Call 72
10.7 Electrocardiography 73
10.8 Spirometry 73
10.9 DNase Concentration Assessment 73
10.10 Sweat Test 74
10.11 DNA Probe Assay 74
10.12 Laboratory Tests 74
10.12.1 Complete Blood Count 74
10.12.2 Biochemistry 75
10.12.3 Serological Tests 75
10.12.4 Assay of Anti-Drug Antibodies, Including Production of Neutralizing Antibodies 76

10.12.5 Urine Express Pregnancy Test 76
10.12.6 Urinalysis 76
10.12.7 Receipt, Preparation, Storage, and Shipment of Biological Samples 76
10.13 Patient Diary 76
10.14 St. George's Respiratory Questionnaire 77
11 EFFICACY ASSESSMENT 77
11.1 Primary Endpoint 78
11.2 Secondary Endpoints 78
12 PHARMACOKINETICS EVALUATION 78
12.1 Pharmacokinetic endpoints 78
13 SAFETY AND TOLERANCE ASSESSMENT 78
13.1 Safety Assessment Parameters 78
13.2 Adverse Events 79
13.2.1 Definitions of AEs 79
13.2.1.1 Adverse Event 79
13.2.1.2 Adverse Reaction 80
13.2.1.3 Serious Adverse Event and/or Serious Adverse Reaction 80
13.2.2 Pregnancy 81
13.2.3 AE Reporting 82
13.2.4 Assessment of AE Severity 82
13.2.5 Causality with the Study/Reference Product 83
13.2.6 Outcomes of the AEs 84
13.2.7 Measures Taken to Reverse AEs 84
13.2.8 Special Events to be Reported to the Sponsor 85
13.2.9 Reporting of SAE/Pregnancy 85
13.2.10 Monitoring of AEs over Time 86
13.2.11 Expedited Reporting 86
13.2.12 Emergency Procedures 86
13.2.13 Clinically Significant Deviations of Laboratory Parameters and Vital Signs 86
14 STOPPING CRITERIA 87

15 DATA ANALYSIS AND STATISTICAL METHODS 87
15.1 Statistical Methods 87
15.2 Subset Calculation 90
15.3 Deviation from the Statistical Data Analysis Plan 91
15.4 Adverse Events 91
16 SOURCE DATA, DATA HANDLING, MAINTENANCE, ARCHIVING, AND RECORD RETENTION 92
16.1 Record Archiving and Retention 93
17 ETHICAL ASPECTS OF THE STUDY 93
17.1 General Provisions 93
17.2 Procedure for Informed Consent Obtaining 93
17.3 Study Subjects Confidentiality and Identification...94
17.4 Recruitment of Subjects from Specific and Vulnerable Populations 94
18 STUDY MONITORING AND STUDY QUALITY ASSURANCE.....................................94
19 PROTOCOL AMENDMENT, DEVIATION, AND VIOLATION...95
20 STUDY FINANCIAL AND ADMINISTRATIVE CONTEXT 96
21 CONFIDENTIALITY AND PUBLICATION ISSUES**ОШИБКА! ЗАКЛАДКА НЕ ОПРЕДЕЛЕНА.**
21.1 General Provisions ....**Ошибка! Закладка не определена.**
21.2 Publications and Use of Study Findings ....**Ошибка! Закладка не определена.**
22 APPENDIX 1. STUDY PROCEDURE SCHEDULE...97
23 APPENDIX 2. QUESTIONNAIRE FOR PATIENTS...100

List of Tables

Table 1. Expected magnitude of differences for the primary efficacy endpoint (ΔFEV_1), which can be detected with the specified subset size ....**Ошибка! Закладка не определена.**

Table 2. Adverse effects occurring in more than 3% of patients with cystic fibrosis during clinic studies of dornase alfa 39

Table 3. Expected magnitude of differences for the primary efficacy endpoint (ΔFEV_1), which can be detected with the specified subset size 91

List of Figures

Figure 1. Graphical flowchart of the study 55

ABBREVIATIONS

µg	Microgram
AE	Adverse event
ALT	Alanine aminotransferase
AST	Aspartate aminotransferase
ATS	American Thoracic Society
CF	Cystic fibrosis
CFTR	Cystic fibrosis transmembrane conductance regulator
CFTR	Cystic fibrosis transmembrane regulator
C_{max}	Maximum concentration
COPD	Chronic obstructive pulmonary disease
CRF	Case Report Form
DBP	Diastolic blood pressure
DNA	Deoxyribonucleic acid
DNase	Dornase alfa
ECG	Electrocardiography
EMA	European Medicines Agency
ERS	European Respiratory Society
ESR	Erythrocyte sedimentation rate
FEV_1	Forced expiratory volume in 1 second
FVC	Forced vital capacity
GCP	Good Clinical Practice
GCS	Glucocorticosteroids
GIT	Gastrointestinal tract
GLM	Generalized Linear Model
h	Hour
Hb	Hemoglobin
hCG	Human choriogonadotropin
HFE	Human hemochromatosis protein
HIV	Human immunodeficiency virus
HR	Heart rate
ICH	International Conference on Harmonization of Technical Requirements for Registration of Pharmaceuticals for Human Use
INN	International nonproprietary name
ITT	Intent-to-treat (analysis as per initially allocated treatment)
IUD	Intrauterine device

kg	Kilogram
LD	Lethal dose
LDH	Lactate dehydrogenase
MCC	Mucociliary clearance
MedDRA	The Medical Dictionary for Regulatory Activities
mg	Milligram
mL	Milliliter
MP	Medicinal product
NCI CTCAE	The National Cancer Institute's Common Terminology Criteria for Adverse Events
NYHA	New York Heart Association
PF	Pulmonary function
PK	Pharmacokinetics
PP	Per Protocol (the patient set per Protocol)
rhDNase	Recombinant human deoxyribonuclease
RR	Respiratory rate
SAE	Serious adverse event
SBP	Systolic blood pressure
SGRQ	St. George's Respiratory Questionnaire
SP	Investigational product
SUSAR	Suspected unexpected serious adverse reaction
T_{max}	Time to maximum concentration
U	Unit
UA	Urinalysis
WMA	World Medical Association

PROTOCOL SUMMURY

STUDY TITLE	
**Study title:**	An open-label, prospective, multicenter, randomized comparative study of pharmacokinetics, clinical effectiveness, and safety of Tigerase (GENERIUM JSC, Russia) and Pulmozyme® (F. Hoffmann-La Roche Ltd., Switzerland) as a part of the combined therapy in the patients with cystic fibrosis
**Study protocol:**	DRN-CFR-II/III
**Study sponsor:**	GENERIUM JSC
**Study phase:**	II/III
**Study design:**	An open-label prospective, multicenter, randomized comparative study
**INVESTIGATIONAL PRODUCT AND REFERENCE PRODUCT**	
**Investigational product:**	**Tigerase (**GENERIUM JSC, Russia)<br>**Pharmacotherapeutic group:**<br>Expectorant mucolytic agent<br>**INN:** Dornase alfa<br>Solution for inhalation, 2.5 mL in polymeric ampoules<br>1 mL of the medicinal product contains:<br>**Active ingredient:** Dornase alfa, 1.0 mg (1,000 U)<br>**Excipients:** Sodium chloride; calcium chloride dihydrate; water for injection
**Reference product:**	**Pulmozyme® (**F. Hoffmann-La Roche Ltd., Switzerland)<br>**Pharmacotherapeutic group:**<br>Expectorant mucolytic agent<br>**INN:** Dornase alfa<br>Solution for inhalation, 2.5 mL in polymeric ampoules<br>1 mL of the medicinal product contains:<br>**Active ingredient:** Dornase alfa, 1.0 mg (1,000 U)<br>**Excipients:** Sodium chloride; calcium chloride dihydrate; water for injection
**STUDY PURPOSE AND OBJECTIVES**	
**Study purpose:**	Comparative evaluation of the pharmacokinetics, efficacy, and safety of Tigerase (GENERIUM JSC, Russia) and Pulmozyme® (F. Hoffmann-La Roche Ltd., Switzerland) as a part of the combined therapy in the patients with cystic fibrosis.

**Study objectives:**	1. To compare the effect of the investigational product Tigerase (GENERIUM JSC, Russia) and the reference product Pulmozyme® (F. Hoffmann-La Roche Ltd., Switzerland) on the pulmonary function according to FEV_1 and FVC as a part of the combined therapy in the patients with cystic fibrosis.<br>2. To compare the rate of exacerbations (respiratory tract infections) in CF patients with FEV_1 ≥40% and ≤100% of the proper value treated with Tigerase (GENERIUM JSC, Russia) or Pulmozyme® (F. Hoffmann-La Roche Ltd., Switzerland) as a part of the combined therapy.<br>3. To compare the duration till the the first exacerbation onset (respiratory tract infection) in CF patients with FEV_1 ≥40% and ≤100% of the proper value treated with Tigerase (GENERIUM JSC, Russia) or Pulmozyme® (F. Hoffmann-La Roche Ltd., Switzerland) as a part of the combined therapy.<br>4. To compare the quality of CF patients' life with FEV_1 ≥40% and ≤100% of the proper value treated with Tigerase (GENERIUM JSC, Russia) or Pulmozyme® (F. Hoffmann-La Roche Ltd., Switzerland) as a part of the combined therapy.<br>5. To compare the DNase concentration in the CF patients' sputum with FEV_1 ≥40% and ≤100% of the proper value treated with the investigational product Tigerase (GENERIUM JSC, Russia) or the reference product Pulmozyme® (F. Hoffmann-La Roche Ltd., Switzerland).<br>6. Safety assessment of Tigerase (GENERIUM JSC, Russia) as a part of the combined therapy in CF patients with FEV_1 ≥40% and ≤100% of the proper value.<br>7. To compare the ability of Tigerase (GENERIUM JSC, Russia) and Pulmozyme® (F. Hoffmann-La Roche Ltd., Switzerland) to stimulate the anti-drug antibodies production including the neutralizing antibodies production .

STUDY METHODOLOGY	
**Study population:**	Patients with a confirmed diagnosis of cystic fibrosis with FEV_1 ≥40% and ≤100% of the proper value aged 18 and older.
**Number of subjects:**	The study will include patients aged 18 and older with a confirmed diagnosis of cystic fibrosis.<br>**Number of the screened patients:** 125 patients (considering the dropout at the screening of about 20% of the patients).<br>**Number of the randomized patients:** 100 patients.<br>**Number of patients who completed the study:** not less than 80 patients.
**Study duration:**	**The study duration for each patient** will be about 28 weeks, including:<br>• **Screening period** — up to 3 weeks<br>• **Treatment period** — 24 weeks ± 1 week
**Study design:**	This study is open-label, prospective, multicenter, randomized, and comparative.<br>The study will investigate the pharmacokinetics, clinical effectiveness, and safety of Tigerase (GENERIUM JSC) and Pulmozyme® (F. Hoffmann-La Roche Ltd., Switzerland) as a part of the combined therapy in the patients with cystic fibrosis.<br>The study will comprise screening and treatment periods.
**Screening period:**	**Visit 1. Screening (Week: -3; Day: -21 until Day -1)**<br>After signing the Patient Information Leaflet and the Informed Consent Form all patients will undergo Screening Visit procedures, during which each patient will be assigned a screening number; patient's medical history and demographics will be collected, the chlorides levels in the sweat glands secret will be determined, and the genotype test will be performed (in the absence of the documented cystic fibrosis diagnosis confirmation), blood will be sampled for complete blood count and biochemistry, immunogenicity test, HIV, hepatitis B and C blood tests, urine will be sampled for urinalysis, and human chorionic gonadotropin (hCG) urine test will be done in order to rule out pregnancy (for women with preserved childbearing potential).

	The patients will undergo a physical examination and vital signs evaluation , electrocardiography (ECG), and spirometry. Assessments using St. George's Respiratory Questionnaire, version 2.2 (Russian version of St. George's Respiratory Questionnaire, SGRQ) will also be carried out. After the results of all examinations will be received and the compliance with all inclusion and exclusion criteria will be confirmed, patients will be invited to the study center for randomization and therapy initiation.
**Treatment period:**	On Day 1, the patients will be allocated to one of the two treatment groups by stratified randomization 1:1 for each of the two strata (FEV_1 40–60% or more than 60% to 100% of the proper value). A unique randomization number will be assigned to each patient.<br><br>The active treatment group of the patients (Group I) will be treated with the investigational product Tigerase (GENERIUM JSC), the reference group (Group II) will be treated with the reference product Pulmozyme® (F. Hoffmann-La Roche Ltd., Switzerland).<br><br>During the treatment, patients will be advised to continue the combined therapy that they have been receiving before enrollment to the study including kinesiotherapy.<br><br>During visits to the study center, patients will be given ampoules of Tigerase or Pulmozyme®, depending on the study group to which they have been randomized. The medicinal product will be inhaled daily in the morning using the jet nebulizer/compressor (one ampoule of the drug per day).<br><br>Throughout the treatment period, the study/reference product will be administered daily for 24 ± 1 weeks of the therapy. The patients have to bring the used medicinal product ampoules back to the study center at visits for registration.<br><br>Patient diaries will be given to all patients to record any complaints and symptoms that occur during the treatment, concomitant therapies, as well as inhalation dates, times, and doses. During the visit of the study center, patients will hand in their Diaries to be checked. During the treatment, patients will visit the study center at least 5 times to be subject to study procedures and safety and immunogenicity evaluations. If necessary, especially for safety reasons the Investigator may invite a patient to an unscheduled visit.

	**The dornase alfa *(DNase)* concentration** will be evaluated in the patients' sputum who agreed to participate in the additional part of the study aimed to evaluate DNase concentrations in sputum and investigate the product pharmacokinetics. This test will be conducted once before and twice after the first inhalation of the study/reference product (Visit 2 (Day 1)).<br><br>An additional part of the study investigating the sputum DNase concentrations will include 24 patients (12 patients in Group I treated with Tigerase and 12 patients in Group II treated with Pulmozyme®).<br><br>***Sputum samples will be taken*** 1 hour ± 15 minutes before inhalation of the study/reference product, then 15 ± 5 minutes and 1 hour 45 minutes ± 15 minutes after the end of the inhalation.<br><br>Based on the individual data of the sputum DNase concentrations , the following pharmacokinetic (PK) parameters will be calculated at each time point:<br>• C_{max} = maximum DNase concentration<br>• T_{max} = time to maximum DNase concentration<br><br>If necessary, the other parameters may be calculated.
**SUBJECTS' SELECTION**	
**Inclusion criteria:**	1. Signed Patient Information Leaflet and Informed Consent Form for participation in the study;<br>2. Men and women aged 18 and older;<br>3. Confirmed diagnosis of cystic fibrosis, defined as the combination of the clinical signs and the positive sweat test and/or the detection of 2 CFTR gene mutations by genotype test*;<br>4. FEV_1 is ≥40% and ≤100% of the proper value;<br>5. Participants' ability to understand the study requirements , to give written consent to participate in the study (including using and transferring of the study relevant information on the patient's health), and to follow the procedures specified in the study protocol.<br><br>* If there is a documented confirmation of cystic fibrosis diagnosis, these tests are not required.

Exclusion criteria:	
**Exclusion criteria:**	1. Hypersensitivity to any medicinal product used in this study to their ingredients, as well as significant allergic reactions in history, according to the Investigator's opinion;<br>2. Acute respiratory infection or chronic pulmonary disease exacerbation within 4 weeks prior to Screening and during Screening with or without corticosteroid or antibiotic therapy;<br>3. Comorbiditiesand conditions that, as to the Investigator's opinion, jeopardize the safety of the patient during his/her participation in the study, or which will influence the analysis of safety data if the disease/condition aggravates during the study, including:<br>✓ Severe renal malfunction (serum creatinine is more than 1.5 times higher than the upper limit of normal);<br>✓ Severe hepatic malfunction (serum activity of alanine aminotransferase (ALT) or aspartate aminotransferase (AST) is 2.5 or more times higher than the laboratory upper limit of normal).<br>4. Lung transplantation in history or transplantation scheduled for the period of this study;<br>5. The presence of anti-HIV antibodies, active viral hepatitis B and/or C, and/or hepatic cirrhosis revealed in the history or identified during the Screening;<br>6. Pregnancy and breastfeeding;<br>7. Refusal of male and female patients with preserved reproductive function to use adequate contraceptive methods* throughout the study and within 30 days after the end of the study/reference product dosing;<br>8. Patients who received blood or blood component transfusions 10 days prior to Screening;<br>9. Drug or alcohol abuse at the time of Screening or in the past, which, as to the Investigator's opinion, makes the patient ineligible for the study;<br>10. Patient’s participation in other clinical studies and/or taking the experimental medicinal product for 30 days before the Screening.<br>* This study considers the following as adequate methods of contraception:<br>➢ Sexual abstinence OR

	➢ Any combination of the following two methods: male or female condom with spermicide, intrauterine device (IUD), diaphragm with spermicide, contraceptive sponge, or cervical cap with spermicide.
**Withdrawal criteria:**	1. Necessity from a medical point of view as to the Investigator's opinion;<br>2. A patient withdraws his/her consent to participate in the study;<br>3. A patient fails to follow the prescribed dosage of the study/reference product and/or the study's procedures (patient's withdrawal must be agreed with a medical monitor);<br>4. Any AE as to the Investigator's opinion makes further participation in the study unfavorable for the patient;<br>5. Development of the conditions requiring the use of medicinal products prohibited by the protocol;<br>6. Patient's death;<br>7. Early termination of the study or the study center closure by the decision of the Sponsor, Investigator, or regulatory authorities.

	**The following reasons require immediate discontinuation of the study/reference product:**<br>1. A patient starts taking another investigational product;<br>2. A patient becomes pregnant;<br>3. Major protocol violations that may affect the safety of the patient and/or integrity of the study data (as agreed with a medical monitor).
**DOSES AND ROUTE OF ADMINISTRATION OF THE STUDY AND REFERENCE PRODUCT**	
**Doses and route of administration of the study and reference product:**	**The investigational product Tigerase** (GENERIUM JSC, Russia) will be inhaled at a dose of 2.5 mg once daily for 24 weeks ± 1 week.<br>**The reference product Pulmozyme®** (F. Hoffmann-La Roche Ltd., Switzerland) will be inhaled at a dose of 2.5 mg once daily for 24 weeks ± 1 week.
**THE STUDY RESULTS EVALUATION**	
**Primary efficacy endpoint:**	The FEV_1 changing (absolute %) at Week 24 ± 1 week versus baseline in the patients of the active treatment group (Tigerase) and the reference group (Pulmozyme®).
**Secondary efficacy endpoints:**	Secondary efficacy endpoints include comparison of the active treatment group (Tigerase) and the reference group (Pulmozyme®) by the following parameters:<br>• FVC changing (absolute %) at Week 24 ± 1 week compared to baseline (Screening);<br>• Number of chronic pulmonary disease exacerbations within 24 weeks ± 1 week of therapy;<br>• The number of days before the development of chronic pulmonary disease exacerbation for a period of 24 weeks ± 1 week of therapy;<br>• Body weight changing at Week 24 ± 1 week compared to baseline (Screening);

	• The average score changing of the subscales "Symptoms", "Activity", "Impacts", and the average total score of the St. George's Respiratory Questionnaire, version 2.2, at Week 24 ± 1 week compared to baseline (Screening).
**Pharmacokinetic endpoints:**	• C_{max} = maximum DNase concentration<br>• T_{max} = time to maximum DNase concentration
**Safety evaluation:**	• Incidence and severity of adverse events (AEs) and serious adverse events (SAEs) during the study period, based on subjective complaints, physical examinations, assessment of vital signs, ECG, laboratory and instrumental tests, and patient diaries of the active treatment group (Tigerase) and the reference group (Pulmozyme®);<br>• The anti-drug antibodies level against dornase alfa within 24 weeks ± 1 week of the therapy in the patients of the active treatment group (Tigerase) and the reference group (Pulmozyme®).
**STATISTICAL ASPECTS OF THE STUDY**	
**Statistical methods:**	The study was planned to test the null hypothesis of the equal efficacy of two medicinal products at W24 versus baseline in the compared groups. The sample size of 100 patients (the number of patients who could have been enrolled based on the feasibility result) was determined to allow to detect the difference (δ) of 5% or more in ΔFEV_1 between the groups with the expected standard deviation (σ) of 10%, the power of 80% (Z_β = 0.84) and two-sided α = 0.05.<br>The trial data were analyzed in three patient populations:<br>1) all patients included in the study (FAS population, full analyzes set); this population was also used as a baseline in the analysis of performance parameters;<br>2) all patients who received at least one dose of the study drug were included in the analysis of safety; the safety population was found to be identical to the FAS population;<br>3) additionally, the efficacy parameters were analyzed in all patients who completed the study without significant deviations from the Protocol (PP-population, Per protocol).<br>Descriptive statistics are given for each studied parameter. The two-sided t-test was used to analyze between-group differences for the primary efficacy endpoint in the intent-to treat population, in addition, a 95% confidence interval was provided.

	The t-test was used for FVC analysis at W24 versus the baseline level. The number of chronic pulmonary disease exacerbations within 24 therapy weeks was analyzed with Poisson regression. Fisher's exact test was used to compare the number and proportion of patients with one, two, three or more exacerbations generally and according to FEV1 baseline stratification between two groups. The time to chronic pulmonary disease exacerbation was analyzed both with the Kaplan–Meier method and the Cox regression model. 'Gender', 'Patient's age', 'Number of concomitant diseases' and 'Treatment group' were considered as covariates. The changes of the mean score on 'Symptoms', 'Activity' and 'Impacts' subscales and mean total score of the Questionnaire at W24 compared to baseline were estimated through paired t-test for intra-group comparison and t-test for group-to-group comparison.<br>Descriptive statistics will be provided for each PK parameter (C_{max}, T_{max}). A formal intergroup comparison, if necessary, will be carried out using the nonparametric Mann–Whitney test, since a significant deviation from the normal distribution is expected.<br>Adverse events were coded by the current Medical Dictionary for Regulatory Activities (MedDRA). Group-to-group comparison of categorical parameters was performed by χ^2-test or Fisher's exact test. The Friedman test was used to assess the quantitative parameters changes at W24 compared to baseline. In the case of statistically significant differences, the post-hoc paired comparison was conducted using a paired t-test or Wilcoxon test depending on the data distribution type. Data on the ADA level of to dornase alfa in both groups were presented through descriptive statistics.<br>The statistical analysis was performed with the Stata application software (StataCorp, USA) version 14.<br>○

	A generalized linear model (GLM) will be built to compare the dynamics of these parameters between the groups.<br>Data on the anti-drug antibodies level against dornase alfa within 24 weeks ± 1 week of the therapy in the patients of the active treatment group (Tigerase) and the reference group (Pulmozyme®) will be presented by means of descriptive statistics.
**ETHICAL AND ADMINISTRATIVE CONTEXT**	
	This study will be conducted in accordance with the Protocol, in strict adherence to the ethical principles of the World Medical Association (WMA) Declaration of Helsinki in the latest edition, adopted on the 64th WMA board in 2013, in strict adherenceto the standards described in the Guidelines for Good Clinical Practice (ICH-GCP) of the International Conference on Harmonization of Technical Requirements for Registration of Pharmaceuticals for Human Use (ICH), Federal Law No. 61, “On Medicine Circulation”, as well as to other applicable regulatory requirements of the Russian Federation legislation , and will be started only after its approval by the Ethics Board of the Ministry of Health of the Russian Federation and an independent ethics committee, as well as approval by an authorized federal executive body. Life and health insurance of subjects is provided by the IC Allianz JSC.

1 STUDY RATIONALE

1.1 Introduction

Cystic Fibrosis (CF) is a common hereditary disease with an autosomal recessive way of inheritance, characterized by systemic involvement of exocrine glands of vital organs, mainly of the bronchopulmonary and gastrointestinal systems. The CF course is usually severe, and the prognosis is poor, making this disease a significant medical and social problem. The disease severity and the CF patient survival are primarily determined by the condition of the bronchopulmonary system: over 90% of CF patients die of lung diseases. [51]

In most countries of Europe and Northern America, the incidence of CF varies from 1:2,000 to 1:5,000 of newborns. [4, 12, 34] According to the data of the Research Center for Medical Genetics and the neonatal screening, the incidence of CF in Russia is 1:8,000–12,000 of newborns with considerable regional variations. The incidence among boys and girls is similar. [9]

In recent years, there has been a significant age distribution shift of CF patients (the disease incidence among adults has increased 4 times, and the mean survival has also increased), due to early diagnosis and application of effective treatment approaches. [1, 4, 8, 10, 12] For example, according to the US Registry, in 1969 the CF patients' survival was 14 years, in 1990 — 28 years, in 1996— 31 years, in 2001 — 32 years, in 2009 – 38 years, when earlier the mean life expectancy used to be 5 years maximum andthe disease was referred to fatal diseases of childhood. [8] According to the literature data, in the 1950s the fatality rate among CF patients under 1 year of age was over 60%, but today in the USA over 50% of CF patients being followed up are over 18 years old. [8] In 1998, the age at death median was 24 years and the survival median — 32 years. [52] Today the expected survival means of a patient born in 2007–2008 is about 50 years. [10] A CF patient born in the UK, Canada, and USA in 1996 is guaranteed to survive for 40 years. [45] At the same time, the calculated expected survival mean of patients with cystic fibrosis in the UK is over 50 years for those born in 2000. [34] In the Russian Federation the mean age of followed patients was 10.2 years in 2009. In 2007 the expected survival median of patients in Moscow was 27.3 years, in Saint Petersburg — 23.6 years with a tendency to an increase in the number of adult patients with CF. [8]

The Cystic Fibrosis Patient Registry has been created in Russia to evaluate the prevalence, genetic and clinical polymorphism, to compare approaches to disease treatment. In 2014 it included the data on 2,131 patients whose average age is 12.8±9.7 years, the median age — 10.2 (15.2) years. [16] In 2014 cystic fibrosis was newly diagnosed in 123 patients; 74.8% was diagnosed by neonatal screening; the age of diagnosis confirmation was 3.5±7.9 years, the age median was 0.8 years. [16] The life expectancy of children with CF born in 2014 is 50.96 years. According to the Registry, this criterion has increased from 43 to 51 over the last three years. [16]

The main predictor for CF patients' survival is the pulmonary function parameters. A forced vital capacity (FVC) less than 40% of the proper value leads to the the about 50% of 2-year mortality rate. [12, 45]

CF pulmonary impairment develops due to the mutation in the cystic fibrosis transmembrane regulator gene (CFTR). The CFTR gene regulates sodium (Na) and chloride (Cl+) transport through the cell membrane and is a part of the cAMP-dependent chloride channel. CFTR protein dysfunction in the epithelial cells of the bronchial tree leads to blocking of the chloride ion transport, increases the sodium ion absorption, and disturbs fluid secretion through the apical membrane of epithelial cells. [9, 74] Currently, about 2,000 CFTR gene mutations are described; the most common mutation of the Caucasians is F508del, which in most cases causes the first CF clinical signs in the early childhood and the development of pancreatic insufficiency. [64, 73] In patients of the European part of the Russian Federation, the rate of F508del mutation is 43% in Moscow and Central Russia and 53% in Saint Petersburg and the North-Western Region. [16] Polymorphisms and mutations of the eNOS, MBL2, and HFE1 genes are associated with the severity of the CF pathological process affecting both the bronchopulmonary and gastrointestinal systems, especially in Russian patients with a F508del homozygous mutation, HFE-1 gene relating to a higher survival rate. [7]

CF clinical manifestations are characterized by the polymorphism. The pathological sputum leads to a chronic infectious and inflammatory process in the lower respiratory tract predominantly in early childhood. A vicious circle (obstruction — inflammation — infection — obstruction) forms shortly after the birth of a child with CF; it is characterized by blockage of the small airways with thick sputum and provides favorable conditions for bacterial growth, lung tissue infiltration by neutrophils, neutrophil releasingincluding the DNA (viscous polyanion), and production of increasingly viscous mucus. The pathological process destroys connective tissue framework, forms bronchiolectasis and bronchiectasis, increases hypoxemia, and causes pulmonary hypertension and pulmonary heart disease. [4, 30, 65, 72]

The chronic respiratory infections predominantly caused by P. aeruginosa, S. aureus, and Haemophilus influenzae are the main cause of CF complications. [5–6, 9] Growing up of the CF patient is accompanied by a decreased respiratory function, changing of the respiratory bacterial flora for more aggressive, increasing the rate of respiratory and gastrointestinal complications. [1, 11] As to the CF Patient Registry data in 2014 the respiratory tract microbiology revealed increasing of the Gram-negative bacteria proportionwith increasing age of patients. [16] The rate of respiratory infections caused by Staphylococcus aureus was 54.5%, by P. aeruginosa (chronic infection) was 32.1%, by B. cepacia complex was 7.3%, by Achromobacter sp. was 3.5%, by Stenotrophomonas maltophilia was 3.3%, by other Gram-negative bacteria (including Achromobacter sp.) was 11.8% of cases. [16]

Thickening of pancreatic secretion in CF disturbs the digestion and absorption of nutritious substances in the gastrointestinal tract, primarily of fats and proteins. In approximately 20% of newborns suffering from CF, the violation of sodium, chloride, and water transport in the small

intestine is accompanied by the so-called meconium ileus, which is the blockage of the distal small bowel with abnormally thick, tenacious meconium. [8, 71]

The use of mucolytic agents aimed at liquefaction of bronchial secretion and maintenance of effective clearing of the bronchial tree from viscous sputum in CF facilitates the control of respiratory infections what is one of the main purposes of CF patients' treatment. In addition to the provision of proper nutrition, a decrease of the number of adverse respiratory effects during complex mucolytic therapy allows maintaining the propriate life quality of CF patients as close to that of healthy people as possible.

Currently, two medicinal products got recognition due to a positive effect on mucociliary clearance in CF patients: recombinant human deoxyribonuclease (rhDNase) and hypertonic solution. [64–66] Free DNA contributing to the abnormally high viscosity of sputum is an important CF treatment target of pulmonary CF impairment. [15, 35, 57, 72, 74]

Dornase alfa, recombinant human deoxyribonuclease 1 (rhDNase), hydrolyzes extracellularDNA (viscous polyanion) entering the sputum from destroyed neutrophils, thus, decreasing sputum adhesiveness and viscosity. Dornase alfa is used as symptomatic therapy in combination with standard therapy in CF patients with a forced vital capacity (FVC) of at least 40% of the proper value. Dornase alfa is also used in the treatment of certain chronic pulmonary diseases (bronchiectasis, chronic obstructive pulmonary disease (COPD), and chronic pneumonia) if as to the doctor's opinion the patient can benefit from the mucolytic effect of the product.

Dornase alfa is inhaled at a dose of 2.5 mg daily. The inhalation efficacy of the CF pulmonary impairment treatment has been confirmed in several studies. [28, 32, 62] Daily inhalations of dornase alfa slow down the pulmonary functions deterioration and reduce the number of respiratory disorder exacerbations in CF patients allowing for long-term use. [2, 9, 11–13, 19–21, 67] The conducted studies of the original medicinal product demonstrated the efficacy of dornase alfa in young patients with CF with moderate pulmonary impairment, as well as in adult patients with more severe pulmonary impairment. [12] The medicinal product is well-tolerated by most patients regardless of the severity of pulmonary impairment. [38, 47, 51]

Medicinal product dornase alfa (Pulmozyme®) by F. Hoffmann-La Roche Ltd., Switzerland is a purified solution of recombinant human deoxyribonuclease 1 produced by the pancreatic gland and other tissues. Some special studies revealed extracellular DNA hydrolysis of the purulent patients' sputum caused with dornase alfa and as a result, significant viscoelastic sputum properties reducing and mucociliary clearance (MCC) improving.

Medicinal product Tigerase (dornase alfa, by GENERIUM JSC, Russia) is a biosimilar to the medicinal product Pulmozyme® (F. Hoffmann-La Roche Ltd., Switzerland).

Comparative preclinical studies of medicinal product Tigerase (GENERIUM JSC) and original medicinal product Pulmozyme® (F. Hoffmann-La Roche Ltd., Switzerland) demonstrated no in vitro activity and toxicity differences.

The results of the phase I study (No. KI 39/14 (КИ 39/14)) "Single-center, open-label, randomized study evaluating the safety, tolerability, and pharmacokinetics of the medicinal product Tigerase (GENERIUM JSC, Russia) in comparison with Pulmozyme® (F. Hoffmann-La Roche Ltd., Switzerland) with repeated inhalation at a dose of 2.5 mg in healthy volunteers" have shown good tolerability and favorable safety profile of medicinal product Tigerase, and along with preclinical data it might be concluded a potential beneficial effect of the medicinal product in the CF patients treatment . [14]

This study is aimed to compare the safety and efficacy of medicinal product Tigerase (GENERIUM JSC, Russia) and original medicinal product Pulmozyme® (F. Hoffmann-La Roche Ltd., Switzerland) with the subsequent marketing authorization of medicinal product Tigerase (solution for inhalation) within the Russian Federation.

1.2 Name and Description of the Investigational Product

1.2.1 Physical and Chemical Properties of Active Substance Dornase Alfa

Chemical name: Recombinant human deoxyribonuclease (rhDNase)
Amino acid sequence:
[redacted]
Empirical formula: C1321H1999N339O396S9
Molecular weight: 37,000 Da
Trade name: Tigerase
International nonproprietary name (INN): Dornase alfa
Pharmaceutical dosage form: Inhalation solution
Composition: 1 mL of the medicinal product contains:

Dornase alfa	1.0 mg (1,000 U)
Sodium chloride	8.77 mg
Calcium chloride dihydrate	0.15 mg
Water for injection	q.s. to 1.0 mL

Appearance: Clear colorless or yellowish solution
Pharmacotherapeutic group: Expectorant mucolytic agent
ATC code: R05CB13

1.3 Preclinical and Clinical Study Results

1.3.1 Preclinical Safety Data

1.3.1.1 Pharmacokinetics and Metabolism in Animals

Absorption

The study of systemic absorption of the original medicinal product after administration by inhalation in rats and human primates has found that the medicinal product has a low systemic absorption (less than 15% in rats and less than 2% in primates).

After the aerosol inhalation with a concentration of 10 μg/L for 13–15 min (the average particle diameter of aerosol was 3–4 μm) the rodents received the dose of 0.15 mg/kg (dose for the lower respiratory tract). DNase was found in the lungs immediately after the administration in concentrations 3.04 to 1.93 μg/lungs and 0.65 to 0.1 μg/lungs after 24 hours.

The product absorption after oral administration in rats was insignificant. After a single oral administration of the medicinal product at a dose of 10 mg/kg to rats, the serum samples were collected after 48 hours. The product concentration was lower or slightly higher than the lower limit of quantification (2 ng/mL). [58]

Changes in concentrations of the medicinal products dornase alfa (GENERIUM JSC) and Pulmozyme® (F. Hoffman-La Roche, Switzerland) in the lung tissues and in the blood serum of rats were assessed in the comparative study of subchronic toxicity following inhalation at a dose of 0.2 mg/kg for 28 days. No significant difference was found between the medicinal products dornase alfa (GENERIUM JSC) and Pulmozyme® (F. Hoffman-La Roche, Switzerland), as well as no difference in groups of females and males.

Toxicokinetics of dornase alfa (GENERIUM JSC) and Pulmozyme® (F. Hoffman-La Roche, Switzerland) in rats at the studied doses is comparable. [13]

The pharmacokinetics of the medicinal product in primates was studied with the inhaled concentrations of 0.39 mg/kg, 1.23 mg/kg, and 5.65 mg/kg. No product concentration in the blood serum was recorded (lower than 2 ng/mL) within 312 hours after the inhalation at a minimum dose. A measurable minimum product concentration was found in the serum after 1.23 mg/kg and 5.65 mg/kg inhalation. The maximum recorded concentration was lower than 40 ng/mL and AUC was 765 and 1,387 h/mL for the dose of 5.65 mg/kg. The medicinal product inhalation are suggested to have its low systemic availability. Its bioavailability for rats was 15%, and for monkeys less than 2%.

Rats and monkeys were used to investigate potential systemic accumulation of the medicinal product after multiple inhalations for 6 months. Systemic accumulation of the medicinal product has not been found. Nevertheless, due to possible influence of the antibodies production to the measurement values during the analysis these data should be interpreted with caution, . [49]

Distribution

Studies in rats and monkeys of the original product have shown that following intravenous administration dornase alfa was cleared rapidly from the serum. The initial volume of distribution was similar to serum volume in these studies. [42] Distribution of the medicinal product was studied using I^{125}–labeled rhDNase. The serum drug's concentration after a single inhalation was minimal. The tissues with perfusion (lungs, liver, and kidneys) had radioactive signal . The signal was recorded at 2 and 30 minutes after the product administration. The urinary organs revealed the main part of the signal; undetectable organ accumulation was found. Four hours after the administration, there is no detecting of the signal as in vivo the radioactive label splits out of the DNase molecule. For this reason, the obtained data should be interpreted with caution. [41]

Metabolism

Dornase alfa is expected to be metabolised by proteases present in all biological fluids. [58] The obtained data in rats suggest that the human serum DNase forms binary complexes with actin or ternary complexes of actin, vitamin D, and DNase. At high doses of DNase, these complexes become saturated, and free DNase leaves them more easily. Apparently, in this case all DNase is in a bound, inactive form. [27, 41]

Elimination

After intravenous administration in rats and primates, the elimination half-life of the medicinal product is 3 to 4 hours. Studies in rats indicate that, following aerosol administration the

disappearance half-life of dornase alfa from the lungs is 11 hours. [42, 58] In intravenous administration, the total clearance was between 17.6 and 58 mL/kg*h, in most tests the medicinal product was eliminated from the circulatory system in 24 hours. The volume of product distribution was approximately equal to the plasma volume (50–60 mL/kg). [68]

1.3.1.2 Toxicity Studies

1.3.1.2.1.1 Acute Toxicity

The acute toxicity study of the original medicinal product has shown that DNase is a well-tolerated product, which is relatively nontoxic at high doses both after inhalation or intravenous administration.

Following a single high doses inhalation to rats and monkeys (it was in the range of 370 to 4,000 μg/kg for both species, which is 2–180 times higher than the doses recommended for humans) no signs of respiratory distress, no effect on feed and water intake or body weight were detected during 2 weeks of follow-up. No signs of immunological reaction were observed either. [31]

No toxic effects of the product were recorded after single intravenous administration to rats and cynomolgus monkeys at a dose of 10 mg/kg. [31]

1.3.1.2.1.2 Irritant Effect

A comparative study of local tolerance was performed as part of the subchronic toxicity study in rats. No significant difference was found between the medicinal products dornase alfa (GENERIUM JSC) and Pulmozyme® (F. Hoffman-La Roche, Switzerland), as well as no difference in groups of females and males.

1.3.1.2.1.3 Multiple-Dose Studies

Intravenous administration of the original product for 2 weeks, at doses up to 1.2 mg/kg, to rats and primates showed no signs of systemic toxicity of the medicinal product [58].

Multiple inhalations of original product dornase alfa to rats and monkeys were performed for 4 to 26 weeks. The administered dose was 201, 766, and 2,176 μg/kg for rats and 247, 527, and 2,153 μg/kg for monkeys, respectively. This exceeded the maximum recommended human dose (5 mg/day) by about 1.3 and 69 times.

No clinical signs of the product toxicity and changes in the organ weights were recorded. Both species demonstrated a dose-dependent elevation in serum antibody titer by the end of the administration period. Elevated antibody titers were observed at the end of the 4-week period in groups of rats receiving maximum and mean doses and in groups of monkeys receiving the maximum dose.

At Week 4 of the study, during the necropsy alveolitis was observed in rats receiving the maximum dose. The condition resolved by the end of the recovery period (in 4 weeks). Pulmonary impairment was not pronounced in rats receiving mean and minimum doses. In primates, one animal out of eight who received the maximum dose showed signs of bronchiolitis. During the necropsy there were lung disease manifestations similar to those in rats. At the end of the recovery period, this condition

resolved. This study demonstrated no correlation between the antibody titer and the severity of the pulmonary impairment. [31]

As a part of a subchronic study test in-house comparative studies of inhalation administration of the medicinal product were conducted in Sprague Dawley rats . Medicinal products (Pulmozyme® (F. Hoffman-La Roche, Switzerland) and Tigerase (Dornase alfa, GENERIUM JSC)) were administered at a dose of 0.2 mg/kg (6.3 times higher than the dose recommended for humans) for 28 days.

The pathomorphological examination was conducted on Day 29 after the first administration of medicinal products (50% of animals in each group) and on Day 57 (the rest of animals). Throughout the study, the animals showed no signs of intoxication, no lethal outcomes were recorded. Physiological parameters and hematological and biochemical blood tests and urinalysis were comparable in experimental and control groups. The pathomorphological examination of internal organs and tissues found no significant differences between the experimental and control groups. The histology in both experimental groups showed the signs of alveolitis immediately after the end of administration (in 58.3% of animals receiving dornase alfa and in 75% of animals receiving Pulmozyme®). However, 28 days after the discontinuation of the medicinal products, the signs of alveolitis were observed at the background level (control level).

1.3.1.2.1.4 Immunogenicity Study

Immunogenicity study analyzed the capability of an investigational product and reference product to form anti-drug antibodies in case of repeated inhalation. High titers of anti-drug antibodies (1/40 and higher) were observed in both males and females in the group of animals treated with dornase alfa, whereas in the Pulmozyme® group, a high titer of anti-drug antibodies was detected only in females.

During the study of toxicokinetics, there was no systemic accumulation of the medicinal product, in the lungs tissues, the drug spreads evenly.

In-house comparative studies of subchronic toxicity and immunogenicity of the medicinal product Pulmozyme® (F. Hoffman-La Roche, Switzerland) and the biosimilar Tigerase (Dornase alfa, GENERIUM JSC) following inhalation administration show no difference regarding toxicity and toxicokinetics and agree with the literature data on the original product Pulmozyme® (F. Hoffman-La Roche, Switzerland).

The animals' condition after inhalation of the medicinal product Dornase alfa (GENERIUM JSC) suggests its good tolerability and safety.

1.3.1.2.1.5 Chronic Toxicity in Young Animals

A toxicity study of the original medicinal product DNase on non-mature animals was performed in 22-day-old rats. The medicinal product was administered at doses of 0, 51,102, and 260 μg/kg/day. The medicinal product was well tolerated by animals; there was no respiratory tract damage (alveolitis, bronchitis). [31, 58]

1.3.1.2.1.6 Carcinogenicity

The carcinogenicity of the original product DNase was studied in rats. Animals received the medicinal product in the aerosol form for 30 minutes/day at doses of 51, 101, and 246 µg/kg/day for 2 years. The maximum dose exceeded the maximum recommended dose for humans by 11–16 times (depending on age). According to the study findings, there was no increase in the incidence of tumors in the experimental groups compared to the control. [58]

1.3.1.2.1.7 Genotoxicity and Mutagenicity

The Ames test with 6 different bacterial strains (4 of *S. typhimurium* and 2 of *E. coli)* at a concentration up to 5,000 µg/plate, a cytogenetic test for chromosomal aberrations of human peripheral blood lymphocytes in concentrations up to 2,000 µg/plate, assay for murine lymphoma at concentrations up to 1,000 µg/plate (with/without metabolic activation) showed no evidence of genotoxicity and mutagenic potential in dornase alfa. [31]

The in vivo product test (micronucleus test) for the damaging effect on the chromosomes of mouse bone marrow cells, with intravenous administration of the product at doses up to 10 mg/kg/day for two consecutive days, also gave a negative result. [31]

1.3.1.2.1.8 Reproductive Toxicity and Teratogenicity

The study of reproductive toxicity was conducted on rats and rabbits with intravenous administration of the original product of DNase at a dose of 10 mg/kg/day. The systemic effect of the product in this study was 600 times greater than the expected systemic effect for the maximum recommended dose to humans. According to the results of the study, there were neither fertility issues nor damaging effects on the fetus, nor effects on pre- and postnatal development of animals. However, the results of animal studies cannot always accurately predict the reaction of the human body, therefore, caution should be exercised when using dornase alfa in pregnancy.

1.3.1.2.1.9 Product Use during Breastfeeding

Studies in female cynomolgus monkeys during the lactation period showed that intravenous administration of high doses of dornase alfa led to insignificant detection of the medicinal product concentrations in breast milk and the detection of less than 0.1% of the product concentration in blood plasma. [31]

1.3.2 Findings of Phase I Clinical Study of Tigerase

A single-center, open-label, randomized comparative phase I study (No.KI 39/14 (КИ 39/14)) was conducted to assess the safety, tolerability, and pharmacokinetics of Tigerase (GENERIUM JSC, Russia) and Pulmozyme® (F. Hoffman-La Roche, Switzerland) on multiple 2.5 mg dose inhalation in healthy volunteers.

Fifty volunteers enrolled in the study inhaled the medicinal product Pulmozyme® (Group I) or Tigerase (Group II) in accordance with randomization at a dose of 2.5 mg per day for 5 days. The follow-up period of volunteers after the product inhalation was 5 days. The assessment of safety

parameters considered: incidence of adverse events, deviations in physical examination findings; vital signs, laboratory parameters, ECG data, peak flowmetry and spirometry (forced expiratory volume in 1 second (FEV_1) and forced vital capacity (FVC)).

The plasma concentration of dornase alfa was assessed in the study. Since the total level of dornase alfa is composed of the endogenous level and the level of active ingredient dornase alfa of the administered medicinal product, two kinds of PK analysis were conducted at all selected time points: by the total concentration of dornase alfa and after the first administration of the medicinal product net of the baseline level of endogenous dornase alfa.

Following the results of the open-label, prospective, multicenter, randomized comparative study of pharmacokinetics, clinical effectiveness, and safety of Tigerase (GENERIUM JSC, Russia) and Pulmozyme® (F. Hoffmann-La Roche Ltd., Switzerland) as part of combined therapy in patients with cystic fibrosis, it was found that:

- Medicinal product Tigerase is well-tolerated by healthy volunteers on multiple 2.5 mg dose inhalation.
- The intergroup comparison did not reveal statistically significant differences between volunteer groups I (Pulmozyme®) and II (Tigerase) in the incidence of AEs (p=0.200). All AEs, registered in the study: 1 (10.0%) in Group I (Pulmozyme®) and 9 (90.0%) in Group II (Tigerase) were mild. One AE in Group I (Pulmozyme®) (dysphonia) and one AE in Group II (Tigerase) (increased AST) were assessed by the Investigator as possibly related to the medicinal product and resolved without consequences. Other 8 out of 9 AEs (88.9%), registered in volunteers of Group II (Tigerase), mainly laboratory data, had a questionable causality to the investigational product.
- Following the study findings, there were no physical deviations in both groups of the volunteers Statistically significant vital signs changes versus baseline such as HR and body temperature in Group I (Pulmozyme®) and SBP and body temperature in Group II (Tigerase) were similar in terms of changes in all vital signs over time in both groups.• There were no statistically significant differences for the most laboratory parameters in both groups, except for the changes in segmented neutrophils, lymphocytes, and platelets in Group II (Tigerase), as well as alkaline phosphatase and alanine aminotransferase (ALT) in Group I (Pulmozyme®). At the same time, the deviations of the quantitative values of segmented neutrophils, lymphocytes, and platelets from the reference values, revealed in volunteers of Group II (Tigerase), were clinically insignificant. All clinically significant laboratory deviations were recorded as AEs.
- The study data revealed no ECG, spirometry, and peak flowmetry values deviations in both groups of healthy volunteers. A statistically significant increase in the peak expiratory flow rate (PEFR) between visits in both groups of volunteers was accompanied by a similar change in the peak flowmetry values for Group I (Pulmozyme®) and Group II (Tigerase).

- A pharmacokinetics data showed that the mean values of dornase alfa plasma concentrations were almost similar in all measured time points after multiple inhalations of the medicinal product in volunteers of Group II (Tigerase) and Group I (Pulmozyme®). The maximum mean values of dornase alfa concentration were 5.52±2.76 ng/mL in Group I (Pulmozyme®) and 6.25±4.79 ng/mL in Group II (Tigerase).
- No reliable increase of the mean dornase alfa plasma concentration was shown as compared to endogenous levels of dornase alfa assessed before inhalation, which is consistent with the literature data suggesting insignificant systemic absorption of dornase alfa.

Thus, the results of the conducted clinical study have shown good tolerability, favorable safety profile and suggested low systemic absorbance of dornase alfa, which is the active ingredient of medicinal product Tigerase.

1.3.3 Clinical Study of Original Medicinal Product Pulmozyme®

Multiple clinical studies of the original medicinal product Pulmozyme® have shown that rhDNase is an effective therapy for CF patients with mild to moderate pulmonary impairment. The medicinal product improves the pulmonary function and reduces the exacerbations incidence in adults and pediatric patients with CF. In the conducted clinical studies, the mild to moderate severity of pulmonary impairment was determined as the value of forced vital capacity (FVC) ≥40% of the proper value.The optimal therapeutic dose was justified in two double-blind, placebo-controlled phase II trials in clinically stable patients with CF in adults and children. [26] The treatment duration in both studies was 10 days. Ramsey et al. (1993) revealed that the use of rhDNase at a dose of 0.6 mg, 2.5 mg, or 10.0 mg once daily for 10 days resulted in a significant increase in FEV_1 by 10–15% versus placebo. In another study conducted by Ranasinha et al. (1993), the use of rhDNase at a dose of 2.5 mg daily for 10 days resulted in an increase in FEV_1 by 13.5% versus placebo. [61]

The first phase III study of rhDNase was a 24-week multicenter, randomized, double-blind, placebo-controlled, parallel-arm, extended open phase study. [49] In addition to the assessment of pulmonary function, this study also assessed the rhDNase effect on the number of respiratory disorder exacerbations, as well as its effect on the vital signs. The patients were recruited in 51 centers; inclusion criteria were the age of 5 years and older and FVC over 40% of the proper value. The use of rhDNase for 24 weeks in CF patients resulted in aa moderate reduction in the risk of respiratory exacerbations requiring parenteral antibiotics administration, as well as an insignificant improvement of the pulmonary function. In addition, the use of rhDNase resulted in a reduced dyspnea, improvement of general well-being, as well as a decrease in the severity of CF symptoms. All the patients who completed participation in phase III double-blind study continued their participation in the 24-week extended open phase. Positive effects of rhDNase therapy persisted throughout the extended phase. There is no clear consensus regarding the degree of pulmonary function improvement as a reliable clinical response in CF. An average FEV_1 increase by 5.8% correlated with long-term use of rhDNase was accompanied by significant relief of symptoms

caused by respiratory impairment in CF (dyspnea, cough, congestion) and, what is more important, a reduced rate of pulmonary disorder exacerbations.

The use of rhDNase was reported during a similar 96-week randomized, double-blind, placebo-controlled multi-center study with the participation of 574 CF patients with sufficiently preserved pulmonary function. [68] During this 2-year study, the pediatric CF patients with early stage of pulmonary impairment receiving rhDNase showed improvement of pulmonary function and the risk reduction of respiratory impairment exacerbations requiring intravenous administration of antibiotics as compared with the placebo group. For 2 years, in the rhDNase treatment group the pulmonary function remained at the baseline level or was higher. Despite a less severe degree of pulmonary disorder in patients of the study, the risk reduction of disease exacerbations was similar to the risk reduction observed in the previous 6-month study with the patients with FVC >40% of the due value and amounted to 34%. [59] Considering that respiratory disorder exacerbations are probably the main factor for a decrease in pulmonary function, a reduction in the number of such exacerbations should slow down the deterioration of pulmonary function. [23, 58]During a 12-week study assessing the efficacy of rhDNase inhalations in the group of 320 CF patients with pronounced pulmonary impairment (FEV_1 <40% of the proper value), the patients receiving rhDNase showed statistically significant improvement of FEV_1 values versus the placebo group. [58] No significant reduction in the number of pulmonary disorder exacerbations requiring parenteral administration of antibiotics was noted among the patients receiving rhDNase versus the placebo group. Despite a tendency to a reduction of the total number of in-patient treatment cases of observed in the rhDNase treatment group, no significant differences were revealed. This study demonstrates effectivness and safety of the rhDNase in CF patients with pronounced pulmonary impairment. [48]

The long-term effect of rhDNase on the disease progression was studied by Shah et al. [69] during a 4-year controlled study. The patients of both groups were comparable in the degree of pulmonary impairment, age, and sex.

Over 4 years of the study, the number of cases of infection exacerbations per patient a year was higher in the control group versus the rhDNase treatment group. The antibiotics administration (number of days of intravenous administration of antibiotics per patient a year) was also significantly higher in the control group versus the rhDNase treatment group. During the study, there was no statistically significant difference in the proven decrease rate of FEV_1 between the patients of both groups. Nevertheless, the investigators tended to think that long-term treatment with rhDNase leads to slowing down the rate of FEV_1 reduction.

Most studies did not provide even the standard microbiology data; however, a substantial portion of pulmonary disorder exacerbations of CF patients was caused by *Staphylococcus aureus* and *Pseudomonas aeruginosa*. [47] A 12-month study assessed the effect of rhDNase on the presence of pathogenic organisms in lower respiratory airways. [39] In general, the number of cases of positive culture was significantly higher in patients who did not receive treatment than in the treatment group. The most significant difference was found for *S. aureus:* over a 12-month period,

the incidence rate of cases of positive culture in the non-treatment group was 30%, whereas in the treatment group it was 16% ($p < 0.05$). A lower rate of infections may be related to a lower number of respiratory disorder exacerbations and a less pronounced inflammatory process.

During the post-marketing study, Hodson et al. [39–40] reported the results of rhDNase treatment comparing the data of patients who did not receive rhDNase and of those who did. The study assessed the effect of the medicinal product on FEV_1 and the number of exacerbations per year. The FEV_1 analysis included 2,023 patients, and the analysis of disease exacerbations included 4,299 patients. In general, the patients of the non-treatment group showed a decrease of the mean FEV_1value (% of the proper value), which was 1.1 in 1 year and 2.3 in 2 years. At Year 1, the values of FEV_1 of the patients treated with rhDNase were higher than the baseline (+2.5% of the proper value) and remained stable throughout the 2-year period (+0.3% of the proper value). A decrease in the exacerbation rate was more significant in the treatment group (by 25%) versus the non-treatment group. This is equivalent to a decrease of 25 exacerbations cases among 100 patients receiving treatment per year. The highest effect of treatment was observed in younger patients. This finding is consistent with the generally accepted view that starting CF treatment early before chronic inflammation leads to irreversible lung damage may be more effective with long-term drug use. [51]

Since the beginning of the widespread dornase alfa administration in 1992, a large amount of its efficacy and safety information has been accumulated around the world both in CF patients and patients with other pulmonary diseases like chronic bronchitis, Kartagener's syndrome, chronic obstructive pulmonary disease, atelectasis, and bronchiectasis. [25, 56, 63]

Therapeutic indications of dornase alfa for the treatment of CF have been expanding over time. For example, almost 50% of CF patients have chronic rhinosinusitis and/or chronic polyposis, and CT scans of the upper respiratory tract reveal different changes in 100% of patients. These pathologic conditions significantly reduce the quality of patients' lives contributing to a more severe course of CF. The successful use of intranasal drug Dornase alfa for the treatment of chronic rhinosinusitis in CF patients has been reported in the literature since 2005. [22, 37]

In Europe, the rhDNase is approved only for CF patients at the age of 5 and older as registration studies have been conducted only for this age category.

All clinical parameters, including histology, pharmacokinetics, distribution, and data of some pilot efficacy studies suggest that the rhDNase will also benefit CF patients under 5 years old. Conducting placebo-controlled studies of sufficient statistical power in CF patients under 5 is challenging. The analysis of adverse events and laboratory parameters was performed to assess the safety of rhDNase. [29, 33, 53] During phase I studies, the plasma concentrations of rhDNase were determined at several time points after single and multiple inhalations with the escalation of rhDNase dose in healthy volunteers and in CF patients. Herewith, only insignificant concentrations of DNase were found in the blood plasma. No study revealed antibodies to rhDNase. [60] Therefore, it was concluded that the systemic exposure of inhaled rhDNase was insignificant.

A lot of studies of rhDNase have been conducted since its market entry. Having reviewed the data of the Epidemiologic Registry of Cystic Fibrosis (ERCF) [39] containing the data on 13,684 CF patients, Hodson et al. reported on the experience of rhDNase use. In general, a careful analysis of rhDNase safety suggests its good tolerability. Although adverse events are the most common reason for study dropouts, the incidence rate and nature of these AEs were approximately the same in rhDNase groups and placebo groups. The use of rhDNase was associated only with voice change (hoarseness), pharyngitis, and laryngitis. [36, 42, 51]

In the randomized, placebo-controlled comparative study of dornase alfa vs. placebo in patients with FVC of at least 40% of the proper value over 600 patients received dornase alfa once or twice daily for 6 months. The majority of observed adverse events were likely manifestations or complications of the underlying disease. In most cases, these adverse effects were mild, transient, and did not require discontinuation of the medicinal product. Adverse events resulting in complete discontinuation of dornase alfa were observed in a very small number of patients, and the rate of therapy discontinuation was similar in the placebo group (2%) and the dornase alfa group (3%). The adverse effects that were more commonly observed (>3%) in patients with cystic fibrosis treated with dornase alfa versus the placebo group are presented in the Table. [43]

In the randomized, placebo-controlled study in patients with clinically apparent cystic fibrosis (FVC <40% of the proper value), the profile of adverse events was similar to that observed in the studies in patients with mild to moderate cystic fibrosis. The adverse effects that were more common in this study (>3%) in the patients treated with dornase alfa [43] are also presented in the Table (Table 2).

Table 2. Adverse effects occurring in more than 3% of patients with cystic fibrosis during clinic studies of dornase alfa

	**Studies in patients with mild to moderate cystic fibrosis (FVC ≥40%) treated for 24 weeks**			**Studies in patients with clinically apparent cystic fibrosis (FVC <40%) treated for 12 weeks**	
Adverse effect (of any intensity and severity)	Placebo (n=325), %	Dornase alfa 2.5 mg, 4 times a day (n=322), %	Dornase alfa 2.5 mg, twice a day (n=321), %	Placebo (n=159), %	Dornase alfa 2.5 mg, 4 times a day (n=161), %
Voice change	7	12	16	6	18
Pharyngitis	33	36	40	28	32
Rash	7	10	12	1	3
Laryngitis	1	3	4	1	3
Chest pain	16	18	21	23	25
Conjunctivitis	2	4	5	0	1
Rhinitis	The difference in the severity of these adverse effects was <3%.			24	30
Decrease in FVC ≥10%*				17	22
Fever				28	32
Dyspepsia				0	3
Dyspnea (when reported as severe)	The difference in the severity of these adverse effects was <3%.			The difference in the overall number of reports on dyspnea (regardless of the degree of manifestation and severity) was <3%.	

*Only a single change, not reflecting all changes in FVC.

According to the data of the European Registry 1994–2000, the tolerability of dornase alfa in pediatric patients under the age of 5 (3,486 people) is as good as in older patients (10,876 people). [40] Trial treatment of pediatric patients under the age of 5 was associated with the following adverse events: pharyngitis, laryngitis, rhinorrhea, hyperthermia, bronchospasm. During the symptomatic treatment for 5–10 days, the existing symptoms were resolved without medicinal product discontinuation. No other adverse events were revealed during the long-term use of dornase alfa. No long-term effects of product inhalations were revealed either. The safety profile in pediatric patients under the age of 5 and older age group is similar. The tolerability of medicinal product dornase alfa by pediatric patients under the age of 5 is also good. Out of 270 patients at the age under 18 followed up at the Moscow Cystic Fibrosis Center only 2 (<1%) showed persistent intolerability to dornase alfa, manifested as bronchospasm. [7]

Another study involved a retrospective analysis of the medical record data of 112 pediatric patients with a verified cystic fibrosis diagnosis. [40] Sixty-eight of whom were at the age of 3 months to 5 years. Clinical examination and special studies were performed for 1 year. The children used dornase alfa for at least 6 months. The mean age of dornase alfa therapy initiation was 3.2 months; 2 children started the treatment at the age of 1.5 months. The reference group (44 patients) consisted ofof children at the age of 2 years and older. During trial treatment with the medicinal product in 68 enrolled patients under the age of 5, 33 patients experienced the following adverse events: pharyngitis, laryngitis, rhinorrhea, hyperthermia, bronchospasm. The patients with adverse events underwent symptomatic therapy for 5–10 days; the existing symptoms were reversed and did not require discontinuation of the medicinal product. All adverse events were observed at the beginning of the trial treatment with dornase alfa, during the first 2 weeks of therapy. No other adverse events were revealed during the long-term use of the medicinal product. All patients completed the study; no long-term effects of product inhalations were revealed. Thus, the use of dornase alfa throughout the study was characterized by its good tolerability. Earlier A.Yu. Voronkova reported adverse effects in 29.34% of CF patients. Moreover, 8.8% of patients had to discontinue the medicinal product due to irreversibility of of the severe symptoms. [7] According to the study data, discontinuation of medicinal product dornase alfa was required only in 2 cases of 112 (1.8%) medical records analyzed.

Incidence of adverse drug reactions is indicated according to the following classification: very common (≥1/10), common (≥1/100, <1/10), uncommon (≥1/1,000, <1/100), rare (≥1/10,000, <1/1,000), very rare (<1/10,000), unknown (impossible to estimate from the available data).

Adverse reactions associated with the dornase alfa treatment are rare (<1/1,000); in most cases, they are mild, of a temporary character, and do not require dose adjustment.
Eye disorders: conjunctivitis.
Respiratory, thoracic, and mediastinal disorders: voice change, dyspnea, pharyngitis, laryngitis, rhinitis (all of them of non-infectious nature), respiratory infections, including the infections caused by Pseudomonas, increased bronchial secretion.
Gastrointestinal disorders: dyspepsia.
Skin and subcutaneous tissue disorders: rash, urticaria.
General disorders: chest pain (pleuritic/non-cardiogenic), pyrexia, headache.
Effect on the results of laboratory and instrumental tests: reduced parameters of pulmonary function.
Patients who have adverse events coinciding with the symptoms of cystic fibrosis may, as a rule, continue treatment with dornase alfa.

According to the literature, adverse events resulting in complete discontinuation of dornase alfa were observed in a small number of patients, and the rate of therapy discontinuation was similar in the placebo group (2%) and the dornase alfa group (3%). The antibodies to dornase alfa were found in less than 5% of patients; however, none of these patients have developed IgE antibodies. Improvement of pulmonary function parameters was observed even after the developing of antibodies to dornase alfa.

Pharmacokinetics in special populations

There is no information about the pharmacokinetics of dornase alfa in special clinical cases. The use of dornase alfa during pregnancy and breastfeeding has not been studied.

Interaction with other medicinal products

Dornase alfa is compatible with standard medications for treatment of cystic fibrosis: antibiotics, bronchial spasmolytics, digestive enzymes, vitamins, inhaled and systemic glucocorticosteroids, and analgesics.

1.4 Risk–Benefit Ratio Assessment for Study Subjects

The risks accepted by the study subjects due to participation in this study should include the risks associated with the use of the investigational product, risks associated with diagnostic procedures, as well as risks associated with the experimental nature of the study.

The study expectes no negative dynamics in the CF patients with FVC ≥40% associated with the use of the investigational product as part of combined therapy for 24 weeks ± 1 week.

1.4.1 Risks Associated with the Use of the Investigational Product

There is no information on adverse drug reactions (ADR) that occurred during treatment with medicinal product Tigerase. The data obtained in the animal studies showed no effect on the functional condition of the cardiovascular system and breathing.

Post-hoc intergroup comparison following Phase I Clinical Study No. KI 39/14 (КИ 39/14) "Single-center, open-label, randomized study to assess safety, tolerability, and pharmacokinetics of the medicinal product Tigerase (GENERIUM JSC, Russia) in comparison with Pulmozyme® (F. Hoffmann-La Roche Ltd., Switzerland) on multiple 2.5 mg dose inhalation in healthy volunteers" did not reveal statistically significant differences between Group I (Pulmozyme®) and Group II (Tigerase) by the incidence rate of adverse events.

1.4.1.1 Warnings and Precautions for Use

Use with caution:

- during pregnancy
- during breastfeeding
- at an age under 18 years

There is no information on the effect of dornase alfa on the patient's ability to drive or to use machines.

1.4.1.2 Overdose

No cases of overdose with dornase alfa were recorded.
Single inhalations of the medicinal product to animals at doses up to 180 times higher than those commonly used in children were well tolerated. Oral administration of dornase alfa to rats at doses up to 200 mg/kg was also well-tolerated.
According to the literature, in the clinical studies CF patients received up to 20 mg dornase alfa twice a day for 6 days and 10 mg twice a day under a split-course regimen (2-week administration followed by a 2-week break) for 168 days. Both dosing schedules were well-tolerated.

1.4.1.3 Risks Associated with the Use of the Study/Reference Product

The inhalation of the study/reference product using jet nebulizer/compressor shall be performed in strict compliance with the manufacturer's operating and maintenance instructions of the jet nebulizer/compressor.

During the study, the patients' health condition is monitored at the planned visits to the study center and telephone calls. The protocol provides for unscheduled visits allowed by the decision of the Investigator.
At the end of the study, the patients will be advised to continue treatment with dornase alfa medicinal products under the physician's supervision.

1.4.2. Risks Associated with the Study Diagnostic Procedures

Certain risks may be associated with individual study procedures.
Vein puncture and/or catheterization for blood sampling may be painful, and, in rare cases, it may result in blood clotting or thrombophlebitis and/or peripheral nerve damage (numbness). For the prevention of complications, blood sampling shall be performed by qualified healthcare professionals in strict compliance with the rules for similar manipulations.
Discomfort may be caused by diagnostic procedures, including physical examination, height and weight measurement, measurement of vital signs (RR, BP, HR, body temperature).

During electrocardiography (ECG), discomfort may be associated with placement of electrodes onto the skin or their removal (in case of excessive hair growth on the man's chest, the areas of electrode placement will probably have to be shaven).

Spirometry will be performed with the use of regularly calibrated spirometer. The spirometer should issue results in paper and electronic form.

Spirometry is accompanied by substantial tension in respiratory muscles, stress on the chest, increased intrathoracic, intra-abdominal, and intracranial pressure. Test contraindications include aortic aneurysm; pulmonary hemorrhage and blood expectoration; pneumothorax; post-surgical period during one month after abdominal or thoracic surgery; venous valve insufficiency of the lower extremities with metabolic and trophic disturbances, varicose vein disease, a tendency to excessive blood clotting; stroke, myocardial infarction during the last three months; uncontrolled hypertension.

Spirometry is repeated for several times to obtain consistent results, in some cases this may be accompanied by minor fatigue and dizziness of various severity, which resolve within 1–3 minutes.

Spirometry is performed by the clinicians taking part in the study, which will prevent complications when recording the forced expiration spirogram.

The spirometry procedure will conform to the Clinical Guidelines for the Use of Spirometry Method by the Russian Pulmonology Association based on the requirements of the American Thoracic Society (ATS) and European Respiratory Society (ERS). [20]

1.5 Rationale for Reference Product, Administration, and Dosage Schedule

The investigational product is Tigerase (GENERIUM JSC, Russia) for inhalation at a dose of 2.5 mg once daily.

The introduction of the medicinal product Tigerase into the clinical practice, as well as other medicinal products, involves the preliminary safety and efficacy study, which is regulated with several regulatory documents.

The investigational product Tigerase is a biosimilar to medicinal product Pulmozyme®, which has been selected as the reference product of the study. The active ingredient of medicinal products Tigerase and Pulmozyme® is dornase alfa, which isa recombinant human deoxyribonuclease. Medicinal product Pulmozyme® has been used in the clinical practice for over 20 years. Since 1997, the medicinal product has been approved in the Russian Federation for the treatment of patients with cystic fibrosis in combination with standard therapy for pulmonary function improvement.

The conducted clinical studies have clearly demonstratedthe clinical efficacyand safety of medicinal product Pulmozyme® for the symptomatic therapy in combination with standard therapy in patients with a forced vital capacity (FVC) of at least 40% of the proper value.

According to the Guidelines on Expert Evaluation of Medicinal Products [16, 17], the selected reference product have to be original medicinal product; in addition, it have to be approved in the Russian Federation based on its complete Drug Master File. The active ingredient of the biosimilar should be equivalent to the active ingredient of the reference product by its molecular and biological properties. The way of administration, dosing, and pharmaceutical dosage form of a biosimilar should not differ from the reference product.

Medicinal product Pulmozyme® satisfies all the above-mentioned conditions, as it is an original medicinal product with active ingredient dornase alfa that is approved in Russia. The active ingredient and excipients of medicinal product Pulmozyme® are identical to those of medicinal product Tigerase as regards both the composition and concentration. The pharmaceutical dosage form of both medicinal products is also identical.

The data on the original medicinal product Pulmozyme® were used to determine effective doses of the investigational product Tigerase as therapy for cystic fibrosis as well as the medicinal product administration schedule. In accordance with the instructions for medical use of the original medicinal product, Pulmozyme® (dornase alfa) is used once a day at a dose of 2.5 mg (the contents of one ampoule) by inhalation without dilution, using a jet nebulizer. Dornase alfa should not be diluted or mixed with other products or solutions in the jet nebulizer cup. Mixing the product with other medicinal products (MPs) can lead to undesirable structural and/or functional changes in dornase alfa.

Therefore, for the purposes of the clinical study to assess safety and efficacy in patients with cystic fibrosis, the investigational product Tigerase (GENERIUM JSC, Russia) and reference product Pulmozyme® (F. Hoffman-La Roche, Switzerland) will be inhaled once a day at a dose of 2.5 mg (1 ampoule contains 2.5 mg of active ingredient) for 24 weeks ± 1 week.

1.6 Study Quality Statement

This document is the protocol of the clinical study that is planned to be conducted in strict compliance with the Declaration of Helsinki of the World Medical Association, Ethical Principles for Medical Research Involving Human Subjects, dated 1964, as amended; in strict compliance with the standards described in the Guidelines for Good Clinical Practice (ICH-GCP) of the International Conference on Harmonization of Technical Requirements for Registration of Pharmaceuticals for Human Use (ICH); as well as in strict compliance with the applicable legislation of the Russian Federation.

The clinical study will be performed in accordance with the applicable version of the Study Protocol.

The Protocol and any amendments to it, as well as Patient Information Leaflet with the Informed Consent Form, shall be preliminary agreed and approved by the regulatory authorities, the Ethics Board, and an independent ethics committee.

1.7 Study Population

The study will include male and female patients aged 18 and older with the confirmed diagnosis of cystic fibrosis with FEV_1 ≥40% and ≤100% of the proper value who meets all inclusion criteria described in detail in Section 4 of this Protocol.

The diagnosis of cystic fibrosis is defined as the presence of a clinical pattern of the disease in combination with a positive sweat test and/or detection of 2 gene mutations of cystic fibrosis

transmembrane regulator (CFTR) by genotype test (if there is a documented confirmation of cystic fibrosis diagnosis, these tests are not required).

The choice of this population was based on the evidence of pulmonary function improvement and reduced incidence and risk of infectious exacerbations from the clinical studies using dornase alfa in adult CF patients. [12, 71]

Enrollment into the study of the patients with FEV_1 ≥40% and ≤100% of the proper value is based on the data of numerous clinical studies that have demonstrated the efficacy of dornase alfa for the treatment of CF patients with mild to moderate pulmonary impairment. Inhalations of dornase alfa result in the reduction of sputum viscosity and surface adhesiveness in CF. Less viscous sputum is easier cleared away from the lungs with the help of mucociliary clearance and coughing, it improves the pulmonary function and reduces the rate of exacerbations in adult CF patients. The dornase alfa using is safe; the adverse reactions associated with its use are rare. [12, 19, 37–40, 51–52, 58, 69]

The expected number of screened patients is 125 people (including 20% exclusion during the screening).

Number of patients randomized: 100 patients.

Number of patients who completed the study: not less than 80 patients.

The patients will only be enrolled in the study if they have signed the Informed Consent Form, completed all screening procedures, and met all requirements of inclusion/exclusion criteria.

1.8 References

1. Asherova I.K., Kapranov N.I. The Registry as the Instrument to Improve the Quality of Medical Care to Patients with Cystic Fibrosis. Pediatric pharmacology. 2012; 3: 96–100.
2. Asherova I.K., Tarakanova V.V. Recurrent Polysinusitis as a Leading Clinical Sign of Cystic Fibrosis: Successful Endonasal Use of Dornase Alfa. Farmateka. 2010. No. 14. p. 66–8.
3. Bogdanova T.A., Kashirskaya N.Yu., Tolstova V.D., Kapranov N.I. Clinical effectiveness and Safety of Pulmozyme® (Dornase Alfa) in Young Children with Cystic Fibrosis. — Russian Bulletin of Perinatology and Pediatrics (Rossiyskiy Vestnik Perinatologii i Pediatrii). — 2008. No. 4. — p. 35–42.
4. Vakharlovsky V.G., Gembitskaya T.E., Dvorakovskaya I.V., Kruglova V.S., Chermensky A.G., Tsybakova N.Yu., Imyanitov E.N. Cystic Fibrosis: Genetics, Clinical Findings, Pathogenesis, Diagnosis, Treatment, and Prevention. 2008, 35 p.
5. Volkov I.K. Potential for Use of Dornase Alfa (Pulmozyme®) in Pediatric Pulmonology. Pulmonology 2004; 4: 113–117.
6. Volkov I.K. Prospects for the Use of Dornase Alfa in Pediatric Respiratory Medicine. Pulmonology, No. 5, 2011, p. 97–102.

7. Voronkova A.Yu. Clinical effectiveness and Safety of Dornase Alfa in the Treatment of Chronic Bronchopulmonary Process in Children with Cystic Fibrosis: Synopsis of a thesis of the Cand. of Med. Sc. M. 2004; 25.
8. Kapranov N.I. and Kashirskaya N.Yu. Cystic Fibrosis (Present-Day Achievements and Contemporary Issues). Methodological Recommendations. Moscow, 2011.
9. Kapranov N.I. and Kashirskaya N.Yu. Cystic Fibrosis. M.: MEDPRAKTIKA-M. 2014. 672 p.
10. Krasovsky S.A., Chernyak A.V., Amelina E.L. et al. Survival Dynamics of Patients with Cystic Fibrosis in Moscow and Moscow Region in the Periods of 1992–2001 and 2002–2011. Pulmonology 2012; 3: 79–86.
11. Krasovsky S.A. et. al. Role of the Registry of the Moscow Region in the Management of Patients with Cystic Fibrosis. — Pulmonology. — 2013. No. 2, p. 27–32.
12. Monograph on Pulmozyme®, Roche, 2011.
13. Experimental comparative study report on general toxicity, immunogenicity of the local tolerance of the biosimilar medicinal product Dornase Alfa, manufactured by GENERIUM JSC, and the original medicinal product PULMOZYME® (Roche). FSBI 48 CRI of the Ministry of Defense of the Russian Federation. Sergiyev Posad-6, 2015.
14. Report on Clinical Study No. KI 39/14 (КИ 39/14) "Single-center, open-label, randomized study to assess safety, tolerability, and pharmacokinetics of the medicinal product Tigerase (GENERIUM JSC, Russia) in comparison with Pulmozyme® (F. Hoffmann-La Roche Ltd., Switzerland) on multiple 2.5 mg dose inhalation in healthy volunteers".
15. Draft National Consensus. Cystic Fibrosis: Definition, Diagnostic Criteria, Therapy. Current Pediatrics (Voprosy Sovremennoi Pediatrii). 2014; 13 (6): 95–101.
16. Cystic Fibrosis Patient Registry in the Russian Federation. 2014 — M.: MEDPRAKTIKA-M Publishing House, 2015, 64 p.
17. Guidelines on Expert Evaluation of Medicinal Products. Volume I. FSBI SCEEMP. Moscow, 2014.
18. Guidelines on Expert Evaluation of Medicinal Products. Volume IV. FSBI SCEEMP. Moscow, 2014.
19. Simonova O.I., Lukina O.F. Dornase Alfa in Russia: 15 Years Later. Drug Efficacy in Children with Cystic Fibrosis. Current Pediatrics (Voprosy Sovremennoi Pediatrii). 2012; 11(2): 132–138.
20. Federal Clinical Guidelines for the Use of Spirometry Method. Russian Respiratory Society. 2013.
21. Anken ML. Burke W, McDonald G, et al. 1992. Recombinant human DNase inhalation in normal subjects and patients with cystic fibrosis. A phase 1 study. JAMA. 267:1947–51.
22. Armstrong DS, Grimwood K, Carzino R. et al. 1995. Lower respiratory infection and inflammation in infants with newly diagnosed cystic fibrosis. BMJ. 310:1571–2.
23. Armstrong DS. Grimwood K. Carlin JB. et al. 1997. Lower airway inflammation in infants and young children with cystic fibrosis. Am J Respir Cut Care Med. 156:1197–204.

australia.com/content/dam/internet/corporate/roche/en_AU/files/miscellaneous/pulmozy meПульмозим-pi.pdf (accessed 29/05/2015).

24. Bilton D. et al. Pulmonary exacerbation: Towards a definition for use in clinical trials. Report from the EuroCareCF Working Group on outcome parameters in clinical trials. Journal of Cystic Fibrosis Volume 10 Suppl 2 (2011) S79–S81.
25. Chah PL. Conway S, Scott SF, et al. 2001. A case-controlled study with dornase alfa to evaluate impact on disease progression over a 4-year period. Respiration, 68:160-4.
26. Cheanick W 1959. Comparison of tracheobronchial secretion in cystic fibrosis of the pancreas and bronchiectasis. Pediatrics, 24:739-45.
27. Chernick WS, Barbero GJ, Eichel HJ 1961. Invitro evaluation of effect of enzymes on tracheobronchial secretions from patients with cystic fibrosis. Pediatrics, 27:589-96.
28. Clinical guidelines for the care of children with cystic fibrosis. 2011. URL: www.rbht. nhs.uk/childrencf
29. Cimmino M, Tardone M, Cavaliere M, et al. Dornase alfa as postoperative therapy in cystic fibrosis sinonasal disease. Arch Otolaryngol Head Neck Surg 2005;131:1097-101.
30. Collins FS 1992. Cystic fibrosis: molecular biology and therapeutic implications. Science. 256:774-9.
31. Dayan A.D. Pharmacological-Toxicological (Expert Report on Recombinant Human Deoxyribonuclease I (rhDNase, Pulmozyme) Hum Exp Toxicol 1994.
32. Dentice R., Elkins M. Timing of dornase alfa inhalation for cystic fibrosis. Cochrane Database Syst. Rev. 2011; 5: CD007923.
33. Desai M, Weller PH, Spenser DA Clinical benefit nebulized human recombinant DNase in Kartagener’s syndrome. Pediatr Pulmonol 1995;20(5):307-08.
34. Dodge JA, Morison S, Lewis PA et al. Cystic fibrosis in the United Kingdom, 1968-1988: incidence, population and survival. Paediatric and Perinatal Epidemiology 1993; 7: 157166.
35. Döring G. Hoiby N 2004. Early intervention and prevention of lung disease in cystic fibrosis: a European consensus.. J Cyst Fibros, 3:67-91.
36. Eiseuberg JD. Aitken ML. Dorkin HL. et al. 1997. Safety of repeated intermittent courses of aerosolized recombinant human deoxyribonuclease in patients with cystic fibrosis. J Pediair. 131:118-24.
37. Fredenksen B. Pressler T. Hansen A. etal. 2006. Effect of aerosolized rhDNase (Pulmozyme) on pulmonary colonization in patients with cystic fibrosis. Acta I’aediatr. 95:11)70 4.
38. Fuchs IU. Borowitz. DS. Christiansen DH. et al. 1994. Effect of aerosolized recombinant human DNase on exacerbations of respirator, symptoms and on pulmonary function in patients with cystic fibrosis. The Pulmozyme Study Group. V Engl J Med. 331:637-42
39. Hodson M, Geddes D. Cystic Fibrosis. Chapman- London. 1995. 439 p.
40. Hodson ME. McKenzie S. Harms UK. et al. 2003. Dornas alfa in the treatment of eystie fibrosis in Europe: a report from the Epidemiologic Registry of Cystic Fibrosis. Pediatr Pulmonal. 36:427-32.

41. Houtmeyers F. Gosselink R. Gayan-Ramircz G, et al. 1999. Effects of drugs on mucus clearance Eur Respir J, 14:452-67
42. Hubbard R. MeElvaney NG. Birrer P. et al. 1992. A preliminary study of aerosoiised recombinant human deoxyribonuclease in treatment of cystic fibrosis. N Engl J Med. 326:812-5.
43. Jones AP. Wallis CE 2003. Recombinant human deoxyribonuclease for cystic fibrosis. Cochrane Database Syst Rev. (3): CD001127.
44. Jones P.W., Quirk F.H., Baveystock C.M. The St.George's respiratory questionnaire. Respir. Med. 1991; 85 (suppl. B): 25-31.
45. Kerem E, Reisman J, Corey M et al. Prediction of mortality in patients with cystic fibrosis. New England Journal of Medicine 1992; 326: 1187–1191.
46. Konstan MW. Hilliard KA. Norvell TM, etal. 1994. Bronchoalveolar lavage findings in cystic fibrosis patients with stable, clinically mild lung disease suggest ongoing infection and inflammation. Am J Respir Cat Care Med. 150:448–54.
47. Konstan MW. Morgan WJ, Butler SM, et al. 2007. Risk Factors For Rate of Decline in Forced Expiratory Volume in One Second in Children and Adolescents with Cystic Fibrosis. J Pediatr. 151:134–9.e 1.
48. Kristensen K. Recombinant human DNase in conditions other than cystic fibrosis. Ugeskr. Laeg. 2010; 172 (8): 616–619.
49. Lieberman J. 1968. Dornase aerosol effect on sputum viscosity in cases of cystic fibrosis. JAMA. 205:3 12–3.
50. Mainz JG, Mentzel HJ, Schneider G, et al. Sinunasal inhalation of dornase alfa in CF. Results of a double-blind placebo-controlled pilot trial. JCysticFibrosis 2008;7:27.
51. McCoy K, Hamilton S, Johnson C, et al. 1996. Effects of 12-week administration of dornase alfa in patients with advanced cystic fibrosis lung disease. Chest. 10:889–95.
52. McKenzie S.G., Chowdhuru S., Strandvik B., Hodson M.E. Dornase alfa is will tolerated: data from the epiodemiologic registry of systic Fibrosis. Pediat Pulmonol 2007; 42: 10: 928—937.
53. Merkus PJFM, de Hoog M, Van Gent R, et al. DNase treatment for atelectasis in infants with severe respiratory syncytial virus bronchiolitis. Eur Respir J 2001;18:734–47.
54. Mogayzel P.J., Naureckas E.T., Robinson K.A. Cystic Fibrosis Pulmonary Guidelines. Am. J. Respir. Crit. Care Med. 2013; 187: 680–689.
55. Penketh ARL, Wise A, Mearns MB et al. Cystic fibrosis in adolescents and adults. Thorax 1987; 42: 526–532.
56. Petersen NT, Hoiby N, Mordhorst CH, et al. 1981. Respiratory infections in cystic fibrosis patients caused by virus, chlamydia and mycoplasma — possible synergism with Pseudomonas aeruginosa. Acta Paediatr Scand, 70:623–8.
57. Potter JL. Spector S, Matthews LW. et al. 1969. Studies on pulmonary secretions. 3. The nucleic acids in whole pulmonary secretions from patients with cystic fibrosis, bronchiectasis, and laryngectomy. Am Rev Respir Dis, 99:909–16.

58. Pulmozyme® Data sheet. – Roche. –p. 1-10
59. Quan JM. Tiddcns H.A. Sy JP, et al. 2001. A two-year randomized, placebo-controlled trial of dornase alfa in young patients with cystic fibrosis with mild lung function abnormalities.J Pediatr. 139:813–20.
60. Ramsey BW. Astley SJ. Aitken ML, et al. 1993. Efficacy and safety of short-term administration of aerosolized recombinant human deoxyribonuclease in patients with cystic fibrosis. Am Rev Respir Dis. 148:145–51.
61. Ranasinha C. Assoufi ß. Shak S. et al. 1993. Efficacy and safety of short-term administration of aerosoiised recombinant human DNase I in adults with table stage cystic fibrosis, Lancet. 342:199–202.
62. Ratjen F, Hartog CM. Paul K. el al. 2002. Matrix metalloproteases in BAL fluid of patients with cystic fibrosis and their modulation by treatment with dornase alpha Thorax. 57:930–4.
63. Regnis JA, Robinson M, Baileu DL. Mucociliary clearance in patients with cystic fibrosis and in normal subjects. Am J Respir Crit Care Med 1994;150:66–71.
64. Riordan JR. Rommens JM. Keren B, et al. 1989. Identefication of the cystic fibrosis gene: cloning and characterization of complementary, DNA Science. 245:1066–73.
65. Riordan JR. Rommens JM. Keren B, et al. 1989. Identefication of the cystic fibrosis gene: cloning and characterization of complementary, DNA Science. 245:1066–73.
66. Robinson M. Hemming AL. Regnis JA, et al. 1997. Effect of increasing doses of hypertonic saline un mucociliary clearance in patients with cystic fibrosis. Thorax. 52: 900–3.
67. Rosenfeld M, Gibson RL. McNamara S, et al. 2001. Early pulmonary infection, inflammation, and clinical outcomes in infants with cystic fibrosis. Pediatr Pulmonal. 32:356–66.
68. Shak S, Capon DJ, Hellmiss R. etal. 1990. Recombinant human Dnase I reduces the viscosity of cystic fibrosis sputum. Proc Natl Acad Sci USA, 87:9188–92.
69. Shah PL, Bush A, Canny GJ et al. Recombinant human DNase I in cystic fibrosis patients with severe pulmonary disease: a short-term, double-blind study followed by six months open-label treatment. European Respiratory Journal 1995; 8: 954–958.
70. Smith A. L., Redding G., Doershuk C., Goldmann D., Gore E., Hilman B., Marks M., Moss R., Ramsey B., Rubio T., Schwartz R. H., Thomassen M. J., Williams-Warren J., Weber A., Wilmott R. W., Wilson H. D., Yagev R. J. Pediatr. 1988
71. Smyth A. R., Bell S. C., Bojcin S., Bryon M., Duff A., Flume P., Kashirskaya N., Munck A., Ratjen F., Schwarzenberg S. J., Sermet-Gaudelus I., Southern K. W., Taccetti G., Ullrich G., Wolfe S. European Cystic Fibrosis Society Standards of Care: Best Practice guidelines. J. Cyst. Fibrosis. 2014; 13 (Suppl. 1): 23–42.
72. Travis J. Structure, function, and control of neutrophil proteinases. Am. J. Med. 1988; 84: 37–41.

73. Yasuda T, Awazu S, Sato W, et al. Human genetically polymorphic deoxyribonuclease: purification, characterization, and multiplicity of urine deoxyribonuclease I. J. Biochem. 1991
74. Zuelzer WW, Newton WA 1949. The pathogenesis of fibrocystic disease of the pancreas. A study of 36 cases with special reference to the pulmonary lesions. Pediatrics. 4:53–69.

2 STUDY PURPOSE AND OBJECTIVES

Study purpose:

Comparative assessment of pharmacokinetics, efficacy, and safety of medicinal product Tigerase (GENERIUM JSC, Russia) and medicinal product Pulmozyme® (F. Hoffmann-La Roche Ltd., Switzerland) as a part of combined therapy in CF patients.

Study objectives:

1. To compare the effect of the investigational product Tigerase (GENERIUM JSC, Russia) and the reference product Pulmozyme® (F. Hoffmann-La Roche Ltd., Switzerland) on the pulmonary function based on FEV_1 and FVC as part of combined therapy in CF patients.
2. To compare the exacerbation (respiratory tract infections) rate in CF patients with FEV_1 ≥40% and ≤100% of the proper value treated with Tigerase (GENERIUM JSC, Russia) and Pulmozyme® (F. Hoffmann-La Roche Ltd., Switzerland) as a part of combined therapy.
3. To compare the duration till the onset of the first exacerbation (respiratory tract infection) in CF patients with FEV_1 ≥40% and ≤100% of the proper value treated with Tigerase (GENERIUM JSC, Russia) and Pulmozyme® (F. Hoffmann-La Roche Ltd., Switzerland) as a part of combined therapy.
4. To compare the quality of life of CF patients with FEV_1 ≥40% and ≤100% of the proper value treated with Tigerase (GENERIUM JSC, Russia) and Pulmozyme® (F. Hoffmann-La Roche Ltd., Switzerland) as a part of combined therapy.
5. To evaluate DNase concentration in the sputum of CF patients with FEV_1 ≥40% and ≤100% of the proper value following inhalation of the investigational product Tigerase (GENERIUM JSC, Russia) and the reference product Pulmozyme® (F. Hoffmann-La Roche Ltd., Switzerland).
6. To evaluate safety of Tigerase (GENERIUM JSC, Russia) as a part of combined therapy in CF patients with FEV_1 ≥40% and ≤100% of the proper value.
7. To compare the anti-drug antibodies rate including the production of neutralizing antibodies following inhalation of Tigerase (GENERIUM JSC, Russia) and Pulmozyme® (F. Hoffmann-La Roche Ltd., Switzerland) ,.

3 STUDY PROCEDURE

3.1 Study Design

The planned clinical study is an open-label, prospective, multicenter, randomized comparative study of phases II and III to assess the pharmacokinetics, clinical effectiveness, and safety of medicinal product Tigerase (GENERIUM JSC, Russia) and Pulmozyme® (F. Hoffmann-La Roche Ltd., Switzerland) as a part of combined therapy in CF patients.

The conducted studies of the original medicinal product Pulmozyme® have proven the efficacy of dornase alfa in young CF patients with moderate pulmonary impairment, as well as in adult patients with more severe pulmonary impairment. Daily inhalations of the medicinal product slow down the deterioration of pulmonary functions and reduce the number of respiratory disorder exacerbations. The medicinal product is well-tolerated by most patients regardless of the severity of the pulmonary impairment.

Medicinal product Tigerase (Dornase alfa, by GENERIUM JSC, Russia) is a biosimilar to the original medicinal product Pulmozyme® (F. Hoffmann-La Roche Ltd., Switzerland).

The planned phase II/III combined study is based on the results of previous preclinical studies of medicinal product Tigerase and phase I clinical study (No. KI-39/14 (КИ-39/14) "Single-center, open-label, randomized study to assess safety, tolerability, and pharmacokinetics of the medicinal product Tigerase (GENERIUM JSC, Russia) in comparison with Pulmozyme® (F. Hoffmann-La Roche Ltd., Switzerland) on multiple 2.5 mg dose inhalation in healthy volunteers" that revealed good tolerability and favorable safety profile of the investigational product, as well as potential efficacy in treatment of CF patients. [14]

3.2 Study Methodology

This study is open-label, prospective, multicenter, randomized, comparative, and parallel-arm.

According to the Screening results, , the patients who meet the inclusion criteria and are without the exclusion criteria will be randomized 1:1 to one of the treatment groups.

Group I is an active treatment group (50 patients), administered the medicinal product Tigerase (GENERIUM JSC, Russia) at a dose of 2.5 mg, once a day by inhalation for 24 weeks ± 1 week.

Group II is a reference group (50 patients), administered the medicinal product Pulmozyme® (F. Hoffmann-La Roche Ltd., Switzerland) at a dose of 2.5 mg, once a day by inhalation for 24 weeks ± 1 week.

An additional study of DNase sputum concentrations will include 24 patients from among the randomized patients (12 patients in Group I treated with Tigerase and 12 patients in Group II treated with Pulmozyme®). The DNase sputum concentration will be assessed once before the first inhalation and twice after the first inhalation of the study/reference product.

During the treatment, patients will be advised to continue the combined therapy that they received before enrollment to the study, including kinesiotherapy.

All patients enrolled in the study undergo the following successive **study periods**:

- **Screening/enrollment in the study** — for 21 days before the first administration of the study/reference product (3 weeks, Days from -21 to -1).
- **Randomization (Day 1)** — confirmed inclusion/exclusion criteria and patient allocation to one of 2 groups of therapy with a unique randomization number.
- **Therapy** — administration of the study/reference product for 24 ± 1 weeks.

The duration of participation of each patient in the study, including the Screening period, will be about 28 weeks.

Upon the study completion, a final report containing the data on all patients enrolled in the study will be submitted to the regulatory authority in due course.

3.2.1 Measures to Minimize Bias

To minimize research biasin the study, a randomization procedure will be applied.

3.2.1.1 Randomization Procedure

Signing the Informed Consent Form at the Screening stage, the patients are assigned a screening number. Conducting all scheduled procedures and meeting the inclusion criteria, the patient will be randomized to one of the treatment groups using the Automated Interactive System (AIS).

The stratified randomization is being used in this study. Depending on the FEV_1baseline, the patient will be allocated to one of two strata: patients with FEV 40–60% of the proper value and patients with the baseline FEV_1 >60% to 100% of the proper value.

Within the two strata, the subjects will be allocated to one of two therapeutic groups at a ratio of 1:1. A unique randomization number will be assigned to each patient. Herewith, there will be no limitation of the stratum size.

Thus, the study is planned with two groups:

Group I is an active treatment group (50 patients), treated with investigational product Tigerase.

Group II is a reference group (50 patients), treated with Pulmozyme®.

The assessment of FEV_1baseline will be performed at the Screening Visit. The randomization procedure and assignment of the randomization number to the patient will be performed at Visit 2 (Day 1) immediately before the study/reference product inhalation.

The registry of screened and randomized patients is maintained at all study centers. The Registration form may be found in the Study Center File. The Investigator shall regularly fill out the form entering necessary data.

In the case of early discontinuation (withdrawal from the study for any reason) of a patient, his/her randomization number shall not be reused. Newly enrolled patients will be assigned new screening and randomization numbers.

3.2.1.2 Blinding Procedure

This study is open label. Investigators and patients will know which therapeutic regimen is being applied. The blinding procedure will be applied to the quality assessment of spirometry results at the Screening Visit, which will be assessed centrally by a certified functional diagnostics specialist who has no access to the patient's personal data, medical history, or clinical manifestations.

3.3 Study Flowchart

The graphical flowchart of the study is presented below (Figure 1).

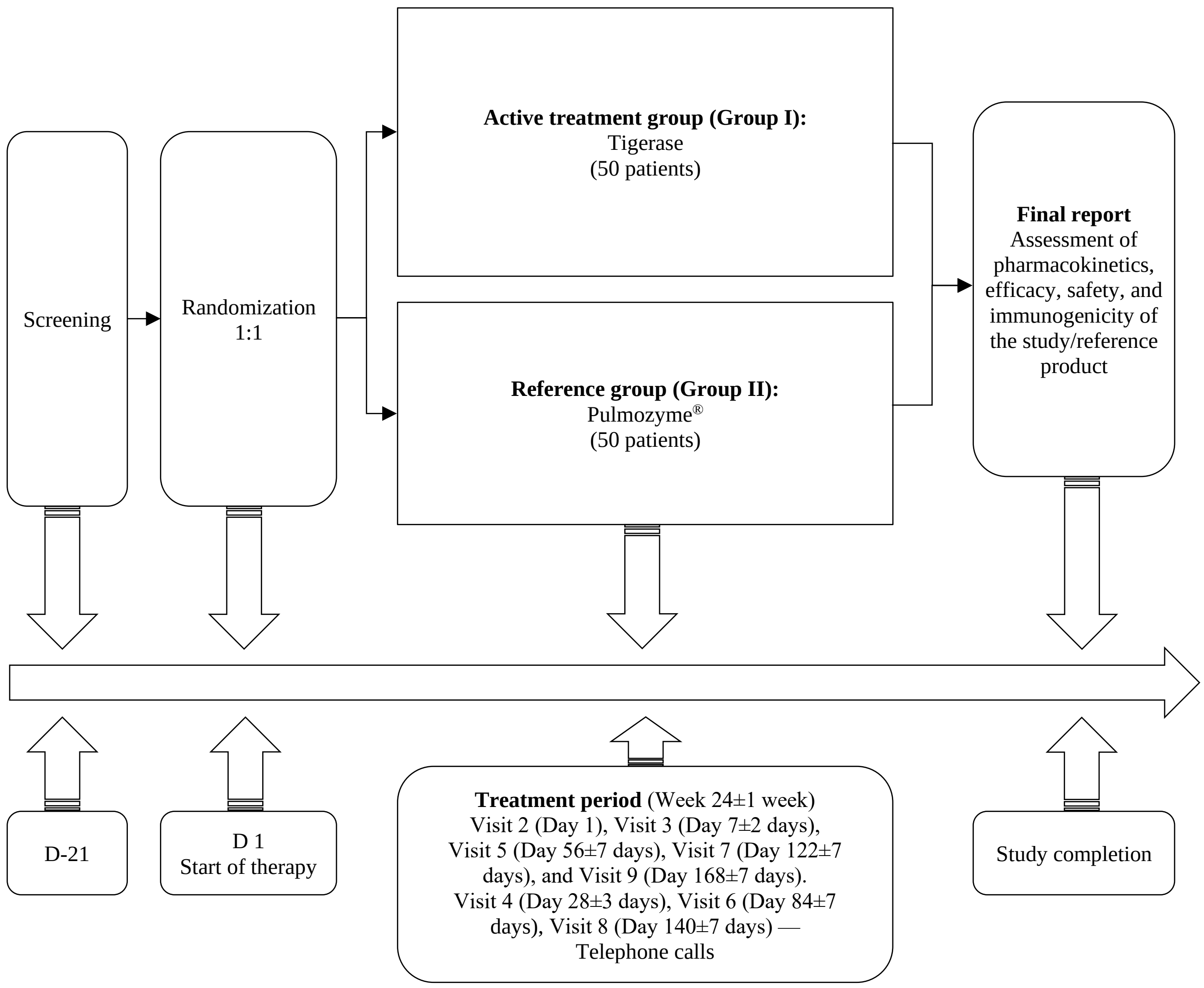

Figure 1. Study flowchart

4 STUDY POPULATION

4.1 Subject Inclusion Criteria

1. Signed Patient Information Leaflet and Informed Consent Form for the study participation ;
2. Men and women aged 18 and older;
3. Confirmed diagnosis of cystic fibrosis, defined as the combination of clinical evidence of the disease and a positive sweat test and/or detection of 2 CFTR gene mutations by genotype test*;
4. FEV_1 is ≥40% and ≤100% of the proper value;
5. Participants' ability to understand the study requirements, to give written consent to participate in the study (including using and transfering of the study relevant information on the patient's health), and to follow the procedures specified in the study protocol.

* If there is a documented confirmation of cystic fibrosis diagnosis, these tests are not required.

4.2 Subject Exclusion Criteria

1. Hypersensitivity to any medicinal product used in this study to its ingredients, as well as a history of significant, according to the Investigator, allergic reactions;
2. Acute respiratory infection or chronic pulmonary disease exacerbation within 4 weeks prior to Screening and on Screening with or without corticosteroid or antibiotic therapy;
3. Comorbidities and conditions that, according to the Investigator, endanger the patient's safety during his/her participation in the study, or which will influence the analysis of safety data if the disease/condition aggravates during the study, including:
   - ✓ Severe renal impairment (serum creatinine is more than 1.5 times higher than the upper limit of normal);
   - ✓ Severe hepatic impairment (the activity of alanine aminotransferase (ALT) or aspartate aminotransferase (AST) in the blood serum is 2.5 or more times higher than the laboratory upper limit of normal).
4. Lung transplantation in history or transplantation scheduled for the period of this study;
5. The presence of anti-HIV antibodies, active viral hepatitis B and/or C, and/or hepatic cirrhosis revealed in the history or identified during the Screening;

6. Pregnancy and breastfeeding;
7. Refusal of male and female patients with preserved reproductive function to use adequate contraceptive methods* throughout the study and within 30 days after the end of the study/reference product dosing;
8. Patients who received blood or blood component transfusions 10 days prior to Screening;
9. Drug or alcohol abuse at the time of Screening or in the past, which, as to Investigator, makes the patient ineligible for the study;
10. Participation of the patient in any clinical studies and/or taking experimental medicinal product for 30 days before the Screening Visit.

* This study considers the following as adequate methods of contraception:

- Sexual abstinence
  OR
- Any combination of the following two methods: male or female condom with spermicide, intrauterine device (IUD), diaphragm with spermicide, contraceptive sponge, or cervical cap with spermicide.

4.3 Premature Withdrawal from Study and Subject Replacement Procedure

4.3.1 Withdrawal Criteria

1. The Investigator considers it medically necessary.
2. A patient withdraws their consent to participate in the study.
3. A patient fails to follow the prescribed dosage of the study/reference product and/or the procedures associated with the study (patient withdrawal must be agreed with a medical monitor).
4. Any AE that, as to the Investigator, makes further participation in the study unfavorable for the patient.
5. Development of conditions requiring the use of medicinal products prohibited by the protocol.
6. Patient's death.
7. Early termination of the study or closure of the study center by the decision of the Sponsor, Investigator, or regulatory authorities.

The following reasons require immediate discontinuation of the study/reference product:

1. A patient starts taking another investigational product.
2. A patient becomes pregnant.

3. Major protocol violations that may affect the safety of the patient and/or integrity of the study data (as agreed with a medical monitor).

4.4 Data Collection and Follow-Up of the Early Withdrawing Subjects

All cases of subject withdrawal from the study will be documented.
The Investigator is responsible for recording the reasons for any premature discontinuation of the study by a patient, as well as for enteringall material details into CFR, with compulsory statement of the reason for dropout.

If the patient's withdrawal was caused by AE/SAE, the Investigator should make every effort to collect the information about the outcome and record the findingsinto the adverse event section of the CRF. If the dropout was caused by SAE, the Investigator should follow the SAE Fast Track procedure to inform the Sponsor .
In the case of early withdrawal of the patient from the study for the reasons described in Section 4.3.1, the procedures shall be performed in accordance with the Protocol (APPENDIX 1. Study Procedure Schedule).

4.4.1 Replacement of Withdrawn Subjects

In case of a dropout of a patient who was included on the basis of screening results, but who discontinued the study prior to the first administration of the study/reference product, a new patient shall be additionally enrolled instead of the excluded. The subjects who prematurely discontinue the study after they started the administration of the study/reference product will not be replaced by new patients, and their data will be taken into account (Section 15.1) in the final analysis. Laboratory tests with erroneous results during Screening may be repeated with the agreement of the Sponsor or his representative Patients who do not meet the study inclusion/exclusion criteria due to some controlled conditions may be re-screened in consultation withthe Sponsor or its representative.

5 INFORMATION ON THE STUDY/REFERENCE PRODUCT

5.1 Pharmaceutical Dosage Form, Composition, and Clinical Pharmacology of the Investigational Product

Trade name: Tigerase

International nonproprietary name (INN): Dornase alfa

Pharmaceutical dosage form: Solution for inhalation

Manufacturer: Generium JSC, Russia

Pharmacotherapeutic group: Expectorant mucolytic agent

ATC code: R05CB13

Composition:

**1 mL of the medicinal product contains:**	
Dornase alfa	1.0 mg (1,000 U)
Sodium chloride	8.77 mg
Calcium chloride dihydrate	0.15 mg
Water for injection	q.s. to 1.0 mL

Appearance: clear colorless or yellowish solution

Packaging and labeling: polymeric ampoules, 8 ampoules in a heat-sealed aluminum foil tape bag placed in a carton. The package has the following text printed with easily readable font in Russian: name of the medicinal product, information on the manufacturer, batch number, manufacturing date, shelf life, strength, volume, potency in units of activity, labeling: "For clinical studies".

Storage conditions: in a dark place at 2°C to 8°C.

5.2 Pharmaceutical Dosage Form, Composition, and Clinical Pharmacology of the Reference Product

Trade name: Pulmozyme®

International nonproprietary name (INN): Dornase alfa

Pharmaceutical dosage form: Inhalation solution

Pharmacotherapeutic group: Expectorant mucolytic agent

ATC code: R05CB13

Composition:

**1 mL of the medicinal product contains:**	
Dornase alfa	1.0 mg (1,000 U)
Sodium chloride	8.77 mg
Calcium chloride dihydrate	0.15 mg
Water for injection	q.s. to 1.0 mL

Appearance: clear colorless or yellowish solution

Packaging and labeling: polymeric ampoules, 6 ampoules in an aluminum foil container placed in a carton. The package has the following text printed with easily readable font in Russian: name of the medicinal product, information on the manufacturer, batch number, manufacturing date, shelf life, strength, volume, potency in units of activity, labeling: "For clinical studies".

Storage conditions: in a dark place at 2°C to 8°C.

5.3 Storage Conditions of the Study/Reference Product

The Investigator or its authorized representative is responsible for the safe and compliant storage of the study/reference product at the study center.

The study/reference product should be retained in the study center at a temperature between 2°C to 8°C, protected from sunlight in a restricted area.

All used ampoules of the study/reference product should be stored at room temperature.

5.4 Accountability of the Study/Reference Product

The Sponsor will provide the study center with the required number of the study/reference products to conduct the study. The relevant quality certificates for the study/reference product will be enclosed in the Investigator's file.

The study/reference product will be given to patients for self-inhaled daily inhalations at a dose of 2.5 mg a day at home using the jet nebulizer/compressor. The used ampoules will be collected for medicinal product registration.

Each stage of receipt, labeling, distribution, and transfer of the study/reference product will be performed and documented in accordance with the applicable clinical trial guidelines and Sponsor’s standard operating procedures.

The responsibility for medicinal product registration in the study center rests withthe Principal Investigator, who may delegate some of the authority for medicinal product registration to the appropriate employee.

The Investigator should ensure that the study/reference product is used in strict compliance with the protocol instructions.

The study/reference product registration should be maintained by the Principal Investigator or his/her designated Responsible Investigatoron specific forms for the recording, storage, and distribution of medicinal products in accordance with the pharmaceutical trial guidelines. The Investigator should keep records of the receipt of the medicinal product, storage conditions, and all procedures with the investigational product at the study center in the Investigator's file.

The study/reference products retained by the Investigator should be stored in closed areas with limited access. The Investigator must keep all used, unused, or partially used packages of the medicinal product until directed by the Sponsor.

5.5 Dosing Schedule of the Study/Reference Product

The investigational product Tigerase/reference product Pulmozyme® is inhaled at a dose of 2.5 mg (which corresponds with the contents of 1 ampoule — 2.5 mL of undiluted solution, 2,500 U) once daily, at the same time without dilutions, using the jet nebulizer/compressor.

The study/reference products are used as a part of combined therapy including chest physical therapy.

The first inhalation of the study/reference product is on the Randomization Day – Day 1.

5.6 Preparation of the Study/Reference Product for Use

Administration of the investigational product Tigerase/reference product Pulmozyme® using the jet nebulizer:

The study/reference product solution in the ampoules intended for single inhalation only should not be diluted or mixed with other medicinal products or solutions in the jet nebulizer cup. Mixing the product with other medicinal products can lead to undesirable structural and/or functional changes in Tigerase/Pulmozyme or another mixture component.

The contents of one ampoule are poured into the jet nebulizer cup equipped with a mouthpiece and a mask. The jet nebulizer is connected to the compressor to create the required airflow (5–8 L/min); a fill volume of the jet nebulizer should be 2–4 mL.

Since dornase alfa applied as a solution through the jet nebulizer enters the lungs during inhaling, the medicinal product should be inhaled through the mouthpiece calmly and evenly. Patients who have difficulties breathing through the mouth during the entire inhalation period using the jet nebulizer may use the jet nebulizer equipped with a tight-fitting mask.

Attention! Ultrasonic nebulizers are not suitable for the administration of the study/reference product as they may inactivate dornase alfa or fail to provide the required aerosol characteristics.

Inhalation procedure:

1. Before performing the procedures described below, wash hands thoroughly.
2. Assemble the jet nebulizer and attach the mouthpiece or respirator according to the manufacturer's instruction.
3. Open the ampoule of the study/reference product and pour the contents into a special jet nebulizer cup.
4. Place the mouthpiece between your teeth and close your lips tightly around it (when using a respiratory mask, put it on so that it fits tightly on the face).
5. Switch on the compressor.
6. Start breathing deeply and slowly, holding your breath for 2 seconds after inhaling, exhale through the nose (rapid deep breathing may cause dizziness, so interrupt the inhalation for a short time from time to time).
7. The inhalation of the study/reference product continues until the medicinal product is used (prior to intermittent aerosol delivery).
8. Switch off the compressor.
9. Rinse your mouth with boiled water (when using the respiratory mask, rinse your eyes and face with water).
10. Dismantle the jet nebulizer.
11. Wash and dry the system components in accordance with the manufacturer's instruction.

Warning:

- Inhalations are carried out not earlier than 1–1.5 hours after eating or physical activity.
- Keep calm during inhalation; make sure that clothes do not hug your neckor make breathing difficult.
- Before inhalation, there is no need to take expectorants or rinse the mouth with antiseptic solutions (potassium permanganate, hydrogen peroxide, boric acid).

The patients will be advised to follow the manufacturer's instructions for the operation and maintenanceof the jet nebulizer/compressor.

6 PROTOCOL COMPLIANCE

6.1 Measures to Ensure Protocol Compliance

Compliance of the study/reference product prescription scheme with the protocol should be assured by adherence to the preparation rules , compliance with the dosing scheme, and the correct inhalation technique, described in sections 5.2, 5.5, 5.6, respectively.

6.2 Patient Compliance

Compliance with the Investigator's recommendations for the prescribed study/reference product will be checked by the Investigator during discussion with the patient at visits, as well as during the visits regarding the accounting of the returned ampoules of the study/reference product and based on records made in the Patient Diary.

7 CONCOMITANT THERAPY, PROHIBITED MEDICATIONS AND THERAPY METHODS

7.1 Concomitant Therapy

During the study, it is allowed to use drugs for the treatment of concomitant diseases that are not related to prohibited concomitant therapy. All therapies, including drug-free methods that patients received during 30 days prior to enrollment into the study and during the study, should be registered in the relevant sections of the Case Report Form (CRF). The information on the patient's concomitant therapy during the study will be recorded in the CRF.
It is necessary to indicate at least the following: international nonproprietary name and trade name of the medicinal product, route of administration, doses, treatment regimen, and indications.

7.2 Prohibited Medicinal Products

Unauthorized medicinal product groups include:

- Any vaccines
- Any unregistered medicinal products or experimental therapy

7.3 Prohibited Therapy Methods

Prohibited therapy methods include:

- Blood and blood component transfusion– Major surgical interventions (performed under anesthesia and/or in hospital settings)

8 EXAMINATION PLAN AND VISITS

8.1 Patient Enrollment

A patient of legal age is considered enrolled into the clinical study after he/she has consented to participate by signing and putting a date on the Informed Consent Form and the Patient Information Leaflet. Immediately after obtaining informed consent, the Investigator will provide the patient with filled out Compulsory Life and Health Insurance Policy of the patient participating in clinical studies of medicinal preparation certifying the patient's insurance during the entire study period.

8.2 Visit 1. Screening/Enrollment to Study (Days -21 to -1)

Examination of a patient during the Screening period is carried out for his/her qualification for the study. The Screening period will last up to 21 days. The patients meeting the inclusion criteria based on the examination results during the Screening period will be enrolled in the study.

The Screening Visit involves the following procedures:

- Procedure for informed consent obtaining
- Collecting demographics, life history, medical history
- Physical examination
- Measurement of vital signs (BP, HR, RR, t°)
- Anthropometry (body weight and height measurement) *(the height is measured once only at the Screening)*
- Quality of life assessment according to St. George's Respiratory Questionnaire (version 2.2.)
- ECG
- Spirometry *(assessment of baseline FEV_1)**
- Complete blood count
- Biochemical blood analysis
- Sweat test *(if there is a documented confirmation of cystic fibrosis diagnosis, this test is not required)*
- DNA probe assay to detect mutations in CFTR gene *(if there is a documented confirmation of cystic fibrosis diagnosis, this test is not required)*

- Blood test for antibodies to human immunodeficiency virus (HIV) — HIV1 and HIV2 (*results obtained not later than 30 days prior to the Screening Visit are suitable*)
- Blood test for viral hepatitis markers: surface hepatitis B antigen, hepatitis C antibodies *(results obtained no later than 30 days prior to the Screening Visit are suitable)*
- Blood test for anti-drug antibodies (IgG, IgE) to dornase alfa, including neutralizing activity
- Urinalysis
- Pregnancy test — urine test for chorionic gonadotropin with a test-strip *(for women with preserved childbearing potential)*
- Registration of the concomitant therapy
- Evaluation of compliance with inclusion/exclusion criteria

* The quality of the Screening Visit spirometry findings will be assessed centrally by a certified functional diagnostics specialist who has no access to the patient's personal data, medical history, or information on clinical manifestations of the disease. The FEV_1 value obtained from the spirometry of the Screening Visit will be the baseline for the assessment of efficacy parameters.

In the case of ambiguous or doubtful laboratory test results, repeat testing (retesting) can be carried out in agreement with the Sponsor or its representative.

Patients who do not meet all the inclusion/exclusion criteria will not be enrolled in the study. Patients not enrolled in the study due to controlled conditions may be re-screened in agreement with the Sponsor or its representative.

If a patient meets all inclusion/exclusion criteria, he/she will be invited to the study center to undergo the randomization and start the study therapy (Visit 2. Day 1). In this case, the patient will be warned that prior to his/her visit to the study center at Day 1 (Visit 2) he/she should not use Pulmozyme® that is administered as part of the standard medical service.

9 TREATMENT PERIOD

9.1 Visit 2. Day 1. Randomization (Week 0)

A procedure of randomization will be performed at Visit 2. A unique randomization number will be assigned to each patient. Group I patients will receive the investigational product Tigerase, Group II patients — the reference product Pulmozyme®.

During participation in the study, the patients receiving Pulmozyme® as part of theirstandard medical service will be provided by the Sponsor with either investigational product Tigerase or reference product Pulmozyme® according to the randomization results.

At Visit 2, the following study procedures will be carried out:

Prior to patient randomization

- Anthropometry (body weight measurement)
- Physical examination
- Measurement of vital signs (BP, HR, RR, t°)
- Registration of the concomitant therapy

Randomization is the assignment of patients to one of therapeutic groups.

After patient randomization

- Sputum sampling *(to study pharmacokinetics of the study/reference product in patients who signed the consent form to take part in the additional part of the study)*
- Handing over the patient diary
- Product dispensing (according to the randomization)

Inhalation of the study/reference product within the study period, including Day 1, should be performed by a patient at home using a jet nebulizer, except for patients who signed the consent form to take part in the additional part of the study to assess DNase concentration in the sputum to evaluate the pharmacokinetics of the medicinal product. These patients will receive an inhalation of the study/reference product at Visit 2 (Day 1) at the hospital using an individual nebulizer.

Sputum sampling schedule at Visit 2. Day 1:

- 1 hour ± 15 min before the inhalation of the study/reference product *(after the patient's randomization);*
- 15 min ± 5 min after the end of inhalation of the study/reference product;
- 1 hour 45 min ± 15 min after the end of inhalation of the study/reference product.

The information for patients about the preparation of the study/reference product for the use and the inhalation procedure performed at home will be provided in the Patient Diary.

9.2 Visit 3. Day 7 ± 2 days (Week 1)

- Anthropometry (body weight measurement)
- Physical examination
- Measurement of vital signs (BP, HR, RR, t°)
- Registration of the concomitant therapy
- Spirometry
- Assessment of clinical symptoms of chronic pulmonary disease exacerbation
- Product return and accountability
- Product dispensing
- Handing over the patient diary
- Returning and checking the patient diary
- Assessment of exclusion criteria
- AE reporting

9.3 Visit 4. Telephone Call. Day 28 ± 3 days (Week 4)

- Interviewing a patient about the current condition, concomitant therapy, compliance and assessment of clinical symptoms of chronic pulmonary disease exacerbation (Section 10.6)
- Registration of the concomitant therapy
- AE reporting

9.4 Visit 5. Day 56 ± 7 days (Week 8)

- Anthropometry (body weight measurement)
- Physical examination
- Measurement of vital signs (BP, HR, RR, t°)
- Registration of the concomitant therapy
- Spirometry
- Complete blood count
- Biochemical blood analysis
- Blood test for anti-drug antibodies (IgG, IgE), including neutralizing activity
- Pregnancy test — urine test for chorionic gonadotropin with a test-strip *(for women with preserved childbearing potential)*
- Assessment of clinical symptoms of chronic pulmonary disease exacerbation
- Product return and accountability
- Product dispensing
- Handing over the patient diary

- Returning and checking the patient diary
- Assessment of exclusion criteria
- AE reporting

9.5 Visit 6. Telephone Call. Day 84 ± 7 days (Week 12)

- Interviewing a patient about the current condition, concomitant therapy, compliance and assessment of clinical symptoms of chronic pulmonary disease exacerbation (Section 10.6)
- Registration of the concomitant therapy
- AE reporting

9.6 Visit 7. Day 112 ± 7 days (Week 16)

- Anthropometry (body weight measurement)
- Physical examination
- Measurement of vital signs (BP, HR, RR, t°)
- Registration of the concomitant therapy
- Spirometry
- Pregnancy test — urine test for chorionic gonadotropin with a test-strip *(for women with preserved childbearing potential)*
- Assessment of clinical symptoms of chronic pulmonary disease exacerbation
- Product dispensing
- Product return and accountability
- Handing over the patient diary
- Returning and checking the patient diary
- Assessment of exclusion criteria
- AE reporting

9.7 Visit 8. Telephone Call. Day 140 ± 7 days (Week 20)

- Interviewing a patient about the current condition, concomitant therapy, compliance and assessment of clinical symptoms of chronic pulmonary disease exacerbation (Section 10.6)
- Registration of the concomitant therapy
- AE reporting

9.8 Visit 9. Day 168 ± 7 days (Week 24 ± 1 week) (End of Therapy Visit / Early Termination Visit, EOT/ET)

- Anthropometry (body weight measurement)
- Physical examination
- Measurement of vital signs (BP, HR, RR, t°)
- Registration of the concomitant therapy

- Quality of life assessment according to St. George's Respiratory Questionnaire (version 2.2.)
- Assessment of clinical symptoms of chronic pulmonary disease exacerbation
- Spirometry
- ECG
- Complete blood count
- Biochemical blood analysis
- Urinalysis
- Blood test for anti-drug antibodies (IgG, IgE), including neutralizing activity
- Pregnancy test *(for women with preserved childbearing potential)*
- Product return and accountability
- Returning and checking the patient diary
- AE reporting

9.9 Unscheduled Visits

The patients may be invited to an unscheduled visit at any time during the clinical study at the discretion of the Investigator, including for safety reasons if it is necessary to repeat an examination or procedure. During unscheduled visits, the required procedures, including laboratory and instrumental examinations, will be performed by the decision of the Investigator. Unscheduled visits should be registered in the source documentation and the CRF. Unscheduled visits should not interfere with the scheduled visits provided for by the clinical study protocol.

10 STUDY PROCEDURES

No study-related procedures will be carried out before the volunteer signs the Informed Consent Form. The volunteer will be given sufficient time to fully familiarize himself/herself with the objectives, upcoming study procedures, rights and responsibilities of the study subjects, the risks and benefits of participating in the study, and to discuss the study with family members and/or other physicians.

Below there are the procedures performed in this study. The Investigator should assess all clinical, laboratory, and instrumental tests results (normal values, clinically insignificant deviation, and clinically significant deviation).

10.1 Collection of Demographics and Medical History

During the screening, after signing of the Informed Consent Form, the following demographics and medical history data will be collected from all patients:

- Patient's age (date of birth), sex, race;
- Information on the underlying medical condition — the time of onset of the first symptoms, date of diagnosis establishment; report on physical and instrumental examination findings, including the genotype test results and results of the test for chloride level in the sweat gland secretion;
- Information on the treatment of the underlying medical condition — all prior therapy, including drug-free treatment of CF within 1 year before the Screening, including the earlier applied therapies indicating at least the international nonproprietary and trade names of the medicinal product, route of administration, dosing schedules, course of treatment, and indications;
- Information on the previous and current diseases, including surgeries within 1 year before the Screening;
- Administration of medications, currently and within 4 weeks before the Screening Visit;
- Immunization history (within 1 year before the Screening Visit);
- History of allergies;
- Presence/absence of complaints as of the examination date.

At subsequent visits, the following is assessed:

- Presence/absence of complaints;
- Collection of information about AEs from the previous visit;
- Collection of information about concomitant conditions and medications from the previous visit.

When signing the informed consent, all women (with preserved childbearing potential) and men should also provide consent for observing the adequate contraception methods throughout the study. In this study, adequate contraception methods should be considered the use of methods described in Section 4.2.

10.2 Anthropometry

Anthropometry (weight and height measurement) should be performed at the Screening Visit. On Day 1, Day 7 ± 2 days, Day 56 ± 7 days, Day 112 ± 7 days, and Day 168 ± 7 days only weight measurements are taken (APPENDIX 1. Study Procedure Schedule). The body weight will be measured using certified scales, without shoes and outer clothing.

10.3 Physical Examination

Physical examination will be performed at the Screening Visit, during treatment on Day 1, Day 7 ± 2 days, Day 56 ± 7 days, Day 112 ± 7 days, and Day 168 ± 7 days in accordance with the Study Schedule (APPENDIX 1. Study Procedure Schedule) to verify the patient's physical parameters and deviations from them, assess the patient's state dynamics, and to reveal AEs/SAEs. Physical examination includes the evaluation of the patient's general condition and the condition of the following:• skin

- visible mucous membranes

- musculoskeletal system
- subcutaneous fat and lymph nodes
- thyroid gland
- respiratory system
- cardiovascular system
- abdominal cavity organs
- urinary system organs
- sensory organs

Clinically significant differences from the initial examination findings should be regarded as AEs and reported accordingly in the source documentation and in the CRF. Clinically significant conditions revealed at the Screening will be recorded as medical history.

10.4 Vital Signs

Vital signs assessment will include measurement of the following:

– systolic blood pressure (SBP)
– diastolic blood pressure (DBP)
– heart rate (per minute) (HR)
– respiratory rate (per minute) (RR)
– body temperature in the armpit (t°)

Vital signs are assessed at the Screening Visit, during treatment on Day 1, Day 7 ± 2 days, Day 56 ± 7 days, Day 112 ± 7 days, and Day 168 ± 7 days (APPENDIX 1. Study Procedure Schedule).

The body temperature should be measured by the same method (axillary temperature). When measuring blood pressure, heart rate, and respiratory rate (after 5 minutes of rest), the patient should be in a sitting or reclining position.

Clinically significant differences from the initial examination findings should be regarded as AEs and reported accordingly in the source documentation and in the CRF.

10.5 Clinical Symptoms of Chronic Pulmonary Disease Exacerbation

Clinical symptoms of chronic pulmonary disease exacerbation will be assessed on Day 7 ± 2 days, Day 56 ± 7 days, Day 112 ± 7 days, and Day 168 ± 7 days (APPENDIX 1. Study Procedure Schedule).

Clinical symptoms of chronic pulmonary disease exacerbation that require therapy with antibiotics in CF patients include the presence of at least 4 out of 12 possible signs or symptoms [24]:

1. Change in the color or amount of sputum
2. Blood expectoration or its aggravation
3. Cough aggravation
4. Dyspnea aggravation
5. Aggravated malaise, fatigue, or atony
6. Temperature above 38°C
7. Anorexia or weight loss
8. Pain or tenderness of nasal sinuses
9. Changed nature of nasal discharge
10. Abnormalities in the chest exam
11. Pulmonary function impairment by 10% or more
12. Radiographic changes

In the case of bronchopulmonary disease exacerbation, the indications for antibiotic therapy, selection and dose of the medicinal product, and therapy duration should be determined by the clinical investigator. The criteria for discontinuation of the antibiotic therapy should be a reversion of basic clinical symptoms of bronchopulmonary disease exacerbation to the level that is a baseline one for this patient. Microbiological analysis of sputum and chest X-ray examination will be performed if required, by the decision of the clinical investigator.

In this study, all cases of chronic pulmonary disease exacerbation will be considered as adverse events of special interest.

10.6 Telephone Call

On Day 28 ± 3 days (Week 4), Day 84 ± 7 days (Week 12), and Day 140 ± 7 days (Week 20), the clinical investigator or employees of the study center authorized by him/her will contact every patient via telephone and ask questions about the state of health, concomitant treatment, medicinal product administration compliance to make sure that all necessary information, including adverse events (AEs) is recorded in the Patient Diary.

To assess clinical symptoms of chronic pulmonary disease exacerbation, the clinical investigator or an employee of the study center authorized by him/her will ask the patient the following questions:

- Do you notice any change in the cough?
- Do you suffer from night cough?
- Do you notice blood in sputum?
- Do you notice the increased volume of sputum and a change in its nature?
- Do you feel that dyspnea is worsening?
- Do you notice a fever or increased heart rate?
- Do you notice poor appetite?
- Do you notice weight loss?
- Do you feel increased fatigue at exertion?

If positive answers are given to these questions, then by the decision of the clinical investigator the patient may be invited to an unscheduled visit to the study center.

10.7 Electrocardiography

A standard 12-lead ECG is performed.

The patient should rest for at least 5 minutes prior to the ECG recording and should be resting during the recording.

ECG is performed at the Screening and on Day 168 ± 7 days during treatment, according to the Study Procedure Schedule (APPENDIX 1. Study Procedure Schedule). General interpretation of ECG should be performed by a certified specialist. The date of ECG registration, patient's surname, and general ECG conclusion should be indicated.

Parameters subject to mandatory recording are the heart rate (HR) and RR, PQ, QRS, and QT intervals. The corrected QTc interval is calculated using Bazett's formula:

$$QTc = QT/\sqrt{(RR(s))},$$

ECG records should be kept in the patient's medical records. The report must include information about whether the ECG is normal, or about the presence of clinically significant or clinically insignificant changes.

If ECG performed during the study reveals any changes that the Investigator considers as clinically significant, the patient, if necessary, should undergo other diagnostic tests in accordance with the local practice and receive appropriate treatment.

10.8 Spirometry

Pulmonary function test (spirometry) will be performed according to the Study Procedure Schedule (APPENDIX 1. Study Procedure Schedule) at the Screening Visit, on Day 7 ± 2 days, Day 56 ± 7 days, Day 112 ± 7 days, and Day 168 ± 7 days in accordance with the standard procedure described in the Guidelines for Conducting of Spirometry.

The quality of spirometry results obtained at the Screening Visit will be assessed centrally by a certified functional diagnostics specialist who has no access to the patient's personal data, medical history, or information on clinical manifestations of the disease. The results of the spirometry performed at the Screening, centrally assessed, and approved, will be used as baseline values.

10.9 DNase Concentration Assessment

The sputum DNase assessment of the patients who have agreed to participate in an additional study to assess the pharmacokinetics of the product will be performed once prior to the first inhalation and twice after the first inhalation of the study/reference product.

Sputum samples will be collected 1 hour ± 15 min before the inhalation of the study/reference product *(after the patient's randomization*), then 15 min ± 5 min and 1 hour 45 min ± 15 min after the end of inhalation (APPENDIX 1. Study Procedure Schedule).

Based on the individual data on DNase concentrations in sputum at each time point, following pharmacokinetic (PK) parameters will be calculated:

- C_{max} = maximum DNase concentration
- T_{max} = time to maximum DNase concentration

If necessary, other parameters may be calculated.

The assessment of DNase concentration in the sputum of the patients will be carried out in the specialized analytic laboratory. The procedure for collection, storage, and transportation of the sputum samples will be described in the laboratory guidelines that will be provided to the study centers. All essential data about biosample collection should be properly documented.

10.10 Sweat Test

The sweat test using the biochemical method (chloride assay in the sweat gland secretion) or sweat test (semi-quantification of sweat chlorides or quantification of sweat electrolytes) will be performed to the patients at the Screening Visit *in the absence of documented confirmation of the cystic fibrosis diagnosis*.

10.11 DNA Probe Assay

DNA probe assay will be performed at the Screening to detect mutations of the cystic fibrosis transmembrane regulator (CFTR) in patients *in the absence of documented confirmation of the cystic fibrosis diagnosis.*

10.12 Laboratory Tests

Blood sampling for complete blood count and biochemistry is carried out on an empty stomachbefore the administration of the study/reference product.

10.12.1 Complete Blood Count

For complete blood count, a venous blood sample will be collected at the Screening, during treatment on Day 56 ± 7 days and Day 168 ± 7 days in accordance with both the Study Procedure Schedule (APPENDIX 1. Study Procedure Schedule) and the standard laboratory procedure of the clinical center.

The following parameters are assessed:

– Hematocrit (HCT)
– Hemoglobin (Hb)
– RBC count
– Platelet count
– Erythrocyte sedimentation rate (ESR)
– Leukocyte count

WBC differential:

– Neutrophils (%)
– Eosinophils (%)
– Basophils (%)
– Monocytes (%)
– Lymphocytes (%)

10.12.2 Biochemistry

A biochemistry test is performed at the Screening, during treatment on Day 56 ± 7 days and Day 168 ± 7 days in accordance with both the Study Procedure Schedule (APPENDIX 1. Study Procedure Schedule), and the standard laboratory procedure of the clinical center.

The following parameters are assessed:

– Total protein
– Glucose
– Total bilirubin
– Urea
– Creatinine
– Alanine aminotransferase (ALT)
– Aspartate aminotransferase (AST)

10.12.3 Serological Tests

– Hepatitis B surface antigen (qualitative test for hepatitis B surface antigen, HBsAg)
– Hepatitis C antigen antibodies (qualitative test for the total anti-HCV)
– Human immunodeficiency virus (HIV1 and HIV2) antibodies (qualitative test for anti-HIV1 and anti-HIV2)

Serological tests are performed on an empty stomach in accordance with the Guidelines for Conducting Laboratory Tests at the Screening for the exclusion of patients with positive test results. Suitable test results should be received not later than 30 days before the Screening Visit.

10.12.4 Assay of Anti-Drug Antibodies, Including Production of Neutralizing Antibodies

Assay of anti-drug antibodies (IgG, IgE), including the production of neutralizing antibodies to the study/reference product, will be performed at the analytical laboratory.
Blood samples will be collected on an empty stomach at the Screening Visit, on Day 56 ± 7 days and Day 168 ± 7 days.

10.12.5 Urine Express Pregnancy Test

Women with preserved childbearing potential (those who have not been surgically sterilized) will undergo a urine test for chorionic gonadotropin with a test-strip (β-hCG) during the Screening Visit, on Day 56 ± 7 days and Day 168 ± 7 days.

Before the treatment initiation, women with preserved childbearing potential should agree to use of reliable contraception methods throughout the study (sexual abstinence or any combination of the following two methods: male or female condom with spermicide, intrauterine device (IUD), diaphragm with spermicide, contraceptive sponge, cervical cap with spermicide).

10.12.6 Urinalysis

Urinalysis (UA) will be performed in accordance with the standard laboratory practice of the clinical center. UA will be performed at the Screening, during treatment on Day 168 ± 7 days in accordance with the Study Procedure Schedule (APPENDIX 1. Study Procedure Schedule).

The following parameters are assessed:

- General properties: specific gravity, pH, protein, glucose, bilirubin, urobilinogen, ketone bodies, nitrites, erythrocytes
- Urinary sediment microscopy: epithelial cells, erythrocytes, leukocytes, cylinders, bacteria, salts

10.12.7 Receipt, Preparation, Storage, and Shipment of Biological Samples

Collection, storage, and transportation of biological samples for laboratory tests should be performed according to requirements and recommendations of the Guidelines for Conducting Laboratory Tests using laboratory kits provided by the Sponsor as part of this study. All essential data about biosample collection should be properly documented.

10.13 Patient Diary

The Patient Diary is the original document filled in by the patient. Before issuing a diary, the Investigator puts down patient information and the date of the next visit in the appropriate section. The patient will record the information about the administration of the investigational product or the reference product (date of administration, time of administration, dose of the medicinal product, batch No.) in the Diary. The patient will record information about complaints that he/she has had since the last visit to the site and concomitant treatment including drug-free modalities (the date the

complaint appeared, the date the complaint resolved, its description, the name of the medicinal product used to relieve the symptoms, the start and end dates of the therapy).

The patient should bring the Diary to each visit. The Diary will be reviewed and assembled by the Investigator, after which the patient will be issued a new diary for use at home. The Patient Diary should be kept at the study center along with the patient's medical records. The Investigator will transfer the information from the patient diary about complaints and concomitant treatment, including drug-free modalities, into an appropriate section of the CRF. The application data of the study/reference product will be used for compliance assessment.

10.14 St. George's Respiratory Questionnaire

St. George's Respiratory Questionnaire (SGRQ), version 2.2, is intended to assess the quality of life of patients with chronic obstructive pulmonary diseases (APPENDIX 2). Filling out the self-administered Questionnaire takes a patient about 15 minutes.

The Questionnaire is composed of 76 questions grouped into 3 parts:

- Part 1 — Symptoms — to measure the degree of anxiety caused by respiratory symptoms.
- Part 2 — Activity — to measure restriction of movement and physical activity.
- Part 3 — Impacts — to assess the current psychosocial consequences of the bronchial obstruction.

In addition, a total score is calculated. The score is determined on the 100-point scale (the higher the value, the greater the impact of the disease is).

A change in the score of 4 points and more was considered clinically significant.

The Investigator will enter the Questionnaire data filled out by the patient at the Screening and on Day 168 ± 7 days into the CRF.

11 EFFICACY ASSESSMENT

Primary and secondary parameters will be used to assess the efficacy of the medicinal product during the study.

11.1 Primary Endpoint

FEV_1 change (absolute %) at Week 24 ± 1 week compared to baseline in patients of the active treatment group (Tigerase) and the reference group (Pulmozyme®).

11.2 Secondary Endpoints

Secondary efficacy endpoints include the comparison of active treatment group (Tigerase) and the reference group (Pulmozyme®) for the following parameters:

- FVC change (absolute %) at Week 24 ± 1 week from baseline (Screening);
- Number of chronic pulmonary disease exacerbations within 24 weeks ± 1 week of therapy;
- The number of days before the development of chronic pulmonary disease exacerbation for a period of 24 weeks ± 1 week of therapy;
- Body weight change at Week 24 ± 1 week from baseline (Screening);
- Change in the average score on the subscales "Symptoms", "Activity", "Impacts", and the average total score of the St. George's Respiratory Questionnaire, version 2.2, at Week 24 ± 1 week from baseline (Screening).

12 PHARMACOKINETICS EVALUATION

12.1 Pharmacokinetic endpoints

- C_{max} = maximum DNase concentration
- T_{max} = time to maximum DNase concentration

13 SAFETY AND TOLERANCE ASSESSMENT

13.1 Safety Assessment Parameters

- Frequency and severity of adverse events (AEs) and serious adverse events (SAEs) during the study period, based on subjective complaints, physical examinations, assessment of vital signs, ECG, laboratory and instrumental tests, and patient diaries of the active treatment group (Tigerase) and the reference group (Pulmozyme®);
- The level of anti-drug antibodies against dornase alfa within 24 weeks ± 1 week of the therapy in patients of the active treatment group (Tigerase) and the reference group (Pulmozyme®).

13.2 Adverse Events

The protocol provides for the recording of all adverse events (AEs) observed after the investigational product or reference product administration to the subject and until the subject completes the study.

Patients participating in the study will be carefully monitored for AEs using general surveying, physical and instrumental examinations, and laboratory tests.

Monitoring will be performed in accordance with the Study Procedure Schedule (APPENDIX 1. Study Procedure Schedule). The adverse events will be classified and entered on the Case Report Form. The classification will be carried out according to the following parameters: seriousness, intensity, causality to the medicinal product, body system, organ and organ system, outcome, actions in relation to the investigational product, and measures taken. The classification will be carried out by the Principal Investigator in accordance with the definitions below.

13.2.1 Definitions of AEs

The following definitions will be used in this Protocol (italics means citation from ICH E2A).

13.2.1.1 Adverse Event

An adverse event (AE) *is any untoward medical occurrence identified in a subject of the clinical study after product administration, which can be either product-associated or have no causality with its administration.*

An adverse event (AE) can be an undesirable and unforeseen symptom (including abnormal laboratory data), an indicator, or a disease, demonstrating a temporal association with the use of the medicinal product, regardless of its association with the medicinal product.

The adverse events can be:

- A new symptom/condition
- A new diagnosis
- Changes in laboratory tests
- Intercurrent diseases and accidents
- Deterioration of health/progression of the disease, which existed before the start of the study
- Disease recurrence
- Increased incidence of exacerbations or intensity of episodic diseases

A concomitant disease or symptoms that existed before the study began will be consideredAE only if their intensity, incidence, or quality changes. Such changes should be reported as an AE by the Principal Investigator. All adverse events (including serious adverse events) should be registered in the Case Report Form.

13.2.1.2 Adverse Reaction

"All noxious and unintended responses to a medicinal product related to any dose should be considered adverse drug reactions. The phrase "responses to a medicinal product" means that a causal relationship between a medicinal product and an adverse event is at least a reasonable possibility, i.e., the relationship cannot be ruled out".

13.2.1.3 Serious Adverse Event and/or Serious Adverse Reaction

A serious adverse event (experience) or reaction is any untoward medical occurrence that at any dose:

- results in death,
- is life-threatening,
- requires inpatient hospitalization or prolongation of existing hospitalization,
- results in persistent or significant disability/incapacity, or
- is a congenital anomaly/birth defect,
- is an important medical event.

Important medical events that are not immediately life-threatening do not lead to fatal outcome or hospitalization but may expose the patient to risk or require interventions aimed at preventing the above-mentioned outcomes can also be classified as SAEs/SARs. Examples of such events can be allergic bronchospasm, convulsions, and neoplasms.

Not to be reported as SAE:

- Hospitalization for social (administrative) reasons in the absence of AE
- Hospitalization for surgery scheduled before the patient's enrollment in the study
- Hospitalization for administration of the study/reference product

Unexpected AR is an AR, that does not match the nature or severity of the relevant product information (e.g., Investigator's Brochure for an unapproved investigational medicinal product or patient's leaflet in the case of an approved medicinal product). This group also includes ARs that are mentioned in the Investigator's Brochure as typical for this class of medicinal products or expected due to the pharmacological properties of the investigational product but not previously observed.

The currently available information on the safety of the investigational product is provided in Section 1.4.1.3, as well as in the Investigator's Brochure.

The Study Sponsor evaluates the expectedness of AR. Information on SUSARs that have arisen during this study will be provided to the Investigator, IEC, and regulatory authorities in accordance with local requirements.

Suspected Unexpected Serious Adverse Reactions (SUSAR) are any serious adverse events that are simultaneously both suspected — that is, (possibly) related to the use of the investigational product — and unexpected and should be regarded as Suspected Unexpected Serious Adverse Reactions (SUSARs).

If the Investigator, upon initial registration of a SAE, determines that there is at least an unlikely causal relationship with the investigational product, and the SAE is unexpected, this event should be recorded as a SUSAR. All SUSARs are subject to expedited reporting to ethics committees and competent regulatory authorities.

13.2.2 Pregnancy

In the case of pregnancy, a study subject shall immediately discontinue the use of the study/reference product.

Pregnancy is not an AE by itself, but it refers to events that require urgent reporting to the Sponsor.

All cases of pregnancy of female study subjects and female partners of male study subjects should be recorded in the original documentation, in the CRF, as well as in the Pregnancy Report Form and reported to the Sponsor within 24 hours from the moment a member of the investigation team learns about the case of pregnancy.

Female subjects participating in the study should be followed up throughout the gestation period and up to 30 days after the pregnancy resolution. Any child who was conceived during the study should also be followed up to 30 days after birth (including pregnancy of the study subject's female partner). The Sponsor should be provided with information on the outcome of pregnancy and the state of the newborn.

Cases of pregnancy of female study subjects and partners of male study subjects should be recorded from the date of signing the Informed Consent Form and until the end of the study.

The Investigator may provide any additional information about the pregnancy and the child only after the pregnant woman provides a consent thereto.

Pregnancy as such is not an adverse event, except when there are reasons to believe that the use of the medicinal products has reduced the effectiveness of contraceptives. Congenital anomalies and malformations in children of study subjects are serious adverse events. Scheduled therapeutic abortions, as well as any serious complications during pregnancy (including spontaneous abortions), should also be reported as serious adverse events. Scheduled therapeutic abortions without complications are not considered AEs.

The outcome of each pregnancy (spontaneous abortion, elective abortion, the birth of a normal child or a child with congenital anomalies or malformations) should be recorded even if the female patient has discontinued participation in the study.

13.2.3 AE Reporting

During each visit, the information about AEs will be collected by the Investigator using open-ended, non-suggestive questions (for example, "How have you been feeling since your last visit?"). For each AE, the Investigator is responsible to collect and register all necessary information in the source documentation and the CRF, including diagnosis or symptoms, the onset and end date, outcome, severity, availability of severity criteria, circumstances that may indicate a possible association with the investigational product or concomitant therapy, underlying diseases or concomitant conditions, study procedures or other causes, actions with respect to the investigational product, drug therapy, medical interventions, the results of laboratory and instrumental tests conducted for the AE, and other circumstances that would enable a comprehensive description of the event in question.

An increase in the severity of an ongoing AE should be regarded as a new AE. The date of a change in severity will be considered the date of a new AE onset, and the previous day is the end date of the initial AE. The date of the SAE onset is the day on which the seriousness criterion occurs. The previous condition, if applicable, should be recorded as a non-serious AE.

If AE is serious, the Investigator should complete the SAE Report Form and send it to the Sponsor within 24 hours from the moment he/she learns about the SAE. New SAE information, which becomes known later, should be recorded, and communicated to the Sponsor in the same way and at the same time. In addition, the Sponsor should be provided with certified copies of the original documents for this SAE (discharge summary, autopsy findings, death certificate, etc.). All information allowing for identification of the patient should be deleted. The documentation should be marked with the study number and patient number.

13.2.4 Assessment of AE Severity

The severity of adverse events reported during this study will be determined in accordance with the Common Terminology Criteria for Adverse Events issued by the National Cancer Institute (NCI CTCAE), v. 4.03.

Each AE will be assigned a severity grade from 1 to 5, where:

The severity of adverse events not listed in NCI CTCAE, v. 4.03, should be assessed according to the following criteria:

- Grade 1. Mild — mild symptoms resulting in minimal discomfort without interfering with daily activities
- Grade 2. Moderate — undesirable event with sufficient discomfort, interfering with normal activities
- Grade 3. Severe — undesirable event, preventing normal daily activities
- Grade 4. Life-threatening — requiring emergency management
- Grade 5. Death — patient's death

13.2.5 Causality with the Study/Reference Product

The Investigator should assess the causality of each adverse event and the study/reference product. The Investigator should assess whether there was a reasonable possibility that the investigational product caused or contributed to the development of the AE in question. In addition to clinical evaluation, the following facts should be considered:

- Temporal association of the event and the use of the study/reference product;
- A reliable mechanism of action of the study/reference product, which could lead to the development of the AE;
- The presence of any other possible cause for the development of the AE;
- Previous reports on similar AEs associated with the study/reference product or other agents in this class;
- AE reappearance after reintroduction of the study/reference product or its resolution after therapy discontinuation, if applicable.

Variants of causality assessment

For each AE, the Investigator should evaluate its possible causality with the study or reference product in accordance with the following categories:

- **Definite**. The adverse event occurred during the period of taking the medicinal product and cannot be explained by concomitant disease or concomitant therapy.
- **Probable.** The adverse event has reasonable time relationship to the investigational product use. Any alternative explanation (concomitant medication, concomitant diseases) of the AE occurrence seems less likely.
- **Possible.** The adverse event may have time relationship to the investigational product use. Any alternative explanations for the causes of the AE, such as concomitant medication or concomitant diseases, are not convincing.

- **Unlikely.** There is a more plausible alternative explanation for the adverse event (for example, the cause of the event was the concomitant medication, concomitant diseases; absence of time relationship with medicinal product intake confirms the doubt).
- **No causality**. The event is not related to the use of the investigational product.
- **Unassessable.** The relationship cannot be established because of insufficient or contradictory information, and the data cannot be supplemented or verified.

13.2.6 Outcomes of the AEs

AEs outcomes are to be reported either at the time of resolution of the AEs, or at the time of study completion for unresolved AEs:

The outcomes of AEs may be classified as follows:

Recovery/resolution	All signs and symptoms of AE disappeared without consequences at the time of the last interview.
In the process of recovery/resolution	The intensity of signs or symptoms decreased by the time of the last interview.
Not resolved	Signs or symptoms did not generally decrease by the time of the last interview.
Recovery/resolution with sequelae	At present, the signs or symptoms of the AE have disappeared, but there are consequences associated with this AE effect.
Fatal outcome	Result in death. If several adverse events occur simultaneously, only the one that directly leads to death can be reported as having resulted in a fatal outcome.
Unknown	The outcome is unknown or unreliable, while the information is not available or cannot be verified.

13.2.7 Measures Taken to Reverse AEs

The measures taken to treat or reduce the effect of an AE as well as measures aimed at eliminating its consequences are defined in accordance with the following categories:

- Not taken: no measures were taken.
- Drug therapy: a new medicinal product has been prescribed, or changes have been made in the dosages of medicines already in use.
- Other: other responsive actions, for example, a surgical intervention.

13.2.8 Special Events to be Reported to the Sponsor

Events that may be subject to the assessment of the study/reference product safety and/or expedited reporting are (including but not limited to):

- Exceeding the dose of the study/reference product;
- Suspected misuse or abuse of the study/reference product;
- Unintended or accidental use of the study/reference product by the person who is not a subject of this clinical study;
- Misuse of medicinal products, including the study/reference product.

These events should be recorded in the CRF, and those of them that are SAEs should be submitted to the Sponsor in accordance with the rules of the SAE reporting.

13.2.9 Reporting of SAE/Pregnancy

All SAEs, regardless of the severity and their relation to the investigational product, must be notified to the Sponsor or its representative within 24 hours from the moment the Investigator receives the information on their occurrence. The SAE data should be submitted in the form of the SAE report. Data on pregnancy should be submitted in the form of a pregnancy report. If necessary, the Investigator may contact the Medical Monitor for advice or clarification.

Reporting of SAE/pregnancy

Company: GENERIUM JSC
E-mail: [redacted]
Fax: [redacted]

The Investigator should instruct each patient to immediately report the occurrence of a SAE or pregnancy during the study or the last visit to the study center. The Investigator will record all available information about the evolving event in the form of a SAE/pregnancy report. In each SAE report form, at least the following data should be indicated: patient information, the name of the event, the seriousness criterion, association with the investigational product, and the outcome at the time of reporting.

For the initial report, the Investigator makes a corresponding entry in the SAE report form, an "Initial report". If additional information about the SAE becomes available (for example, new information about the patient's condition or laboratory test results), the Investigator should complete a new form of the SAE report, indicating it as a "Follow-up report". The original SAE Report Form will be filed in the Site Investigator File.

13.2.10 Monitoring of AEs over Time

The Investigator should follow up all the SAEs until they are resolved. If an AE persisted until the subject completes the clinical study, the Investigator had to record its status as of the study end and ensure its follow-up until it meets one of the following criteria:

- Resolution;
- Stabilization;
- Return to the level observed before the subject was enrolled in the study (if the information is available);
- Evidence of an association between an AE and another pharmaceutical products (other than the investigational product and the reference product) or factors not related to the study;
- Situations where it becomes apparent that further information about the SAE will not be obtained (e.g., the subject or his/her attending physician refuses to provide information, or the subject is lost for follow-up), provided that the Investigator has appropriately requested such information.

13.2.11 Expedited Reporting

The information on the Suspected Unexpected Serious Adverse Reactions (SUSARs) should be submitted to the ethics committee and competent regulatory authority within the established period as this information is subject to expedited reporting.

Operational reporting is done by the Sponsor's authorized employee responsible for safety. For a more detailed description of the pharmacovigilance procedures regarding this study, see the Safety Monitoring Guidelines.

13.2.12 Emergency Procedures

The Investigator is responsible for obtaining information on all emergency medical conditions of patients during the study and providing the necessary medical care in accordance with the effective medical standards. The Patient Information Leaflet and the Informed Consent Form contain the Investigator's contact information. Patients will be advised to contact the Investigator in the event of any emergency conditions during the study.

13.2.13 Clinically Significant Deviations of Laboratory Parameters and Vital Signs

Any clinically significant deviations in laboratory results found during or after the Screening should be attributed to the patient's medical history. After administration of the study or reference product, new or worsening clinically significant laboratory abnormalities should be reported as AEs in the original documentation and in the CRF.

14 STOPPING CRITERIA

The Sponsor or the Principal Investigator has the right to terminate (temporarily or permanently) the study for safety or other reasons (in accordance with Article 40 of the current version of Federal Law No. 61 dated April 12, 2010, On Circulation of Medicines). If such a need occurs, the Sponsor takes actions for timely informing the clinical center about suspension or termination of the study. The Sponsor and the Principal Investigator should also inform the local ethics committee and regulatory authorities in a timely manner. The Principal Investigator may suspend or terminate the study without prior consent of the Sponsor. However, he/she should notify the Sponsor as soon as possibleand provide a written explanation of reasons for suspension or termination of the study. The Investigator should also immediately inform the study subjects about suspension or termination of the study, and, if necessary, provide them with appropriate medical care and follow-up. Terminating the study, the Investigator shall make sure that the interests of the study subjects are duly protected. The study may be terminated by the decision of the authorized federal executive body based on written notification to the head of the study center or the Sponsor.

15 DATA ANALYSIS AND STATISTICAL METHODS

15.1 Statistical Methods

Analysis populations

It is planned that the statistical analysis involves four patient populations:

- All subjects enrolled in the study (**FAS, full analysis set**). This population will be used to describe the basic characteristics, including demographics and anthropometric parameters of patients, data on the underlying and concomitant diseases.
- The safety analysis will include all patients who received at least one dose of the investigational product. This population will also be used to assess primary efficacy parameters. In this case, the patient's group will be determined by the "intention to treat" (**ITT analysis**).
- Additionally, analysis of secondary efficacy parameters will be performed in all subjects who completed the study without any significant Protocol deviations (**PP analysis**).
- The population for the pharmacokinetic analysis will consist of patients for whom data were obtained at least at one time point of the PK measurement plan.

Analysis of the baseline characteristics of subjects

To describe the baseline characteristics of subjects representing numerical variables (age, body weight, laboratory parameters, etc.), the following characteristics will be calculated:

- Number of nonmissing values (N)
- Arithmetic mean (M)
- Standard deviation (SD)
- 95% confidence interval (CI) for the mean
- Median (Med)
- Minimum (Min)
- Maximum (Max)
- Interquartile range (IQR)

The number and proportion of subjects will be provided for qualitative and categorical variables (sex, medical history, the incidence of abnormalities according to physical examination data, etc.).

A comparative intergroup analysis of the patient's baseline characteristics will be performed using the t-test (or Mann–Whitney test) for numerical data and using the χ^2-criterion (or Fisher's exact test) for categorical variables.

Analysis of the primary efficacy endpoint

When analyzing the primary efficacy endpoint (the change in FEV_1 at Week 24 ± 1 week from baseline), the data distribution will be preliminarily tested for normality using the Shapiro–Wilk test, as well as a test for symmetry and kurtosis. In the case of a normal distribution, the intergroup comparison will be performed using a t-test. If there are significant deviations from the normal distribution ($p<0.05$), a non-parametric Mann–Whitney test will be used. The null hypothesis for the equality of the FEV_1 change on Week 24 ± 1 week compared to the baseline between the two groups will be tested with a two-tailed test at a significance level of 0.05 using the Stata14 program. In addition, a 95% confidence interval will be calculated for the difference between the two groups. In addition, the generalized linear model (GLM) including sex, patient age, and medical history as covariates may be used to test the influence of different factors.

Analysis of secondary efficacy endpoints

In this study, the planned secondary efficacy endpoints include the following:

- FVC change (absolute %) at Week 24 ± 1 week from baseline (Screening);
- Number of chronic pulmonary disease exacerbations within 24 weeks ± 1 week of therapy;
- Number of days to a chronic pulmonary disease exacerbation over the period of 24 weeks ± 1 week of therapy;
- Body weight change at Week 24 ± 1 week from baseline (Screening);
- Change in the average score on the subscales "Symptoms", "Activity", "Impacts", and the average total score of the St. George's Respiratory Questionnaire, version 2.2, at Week 24 ± 1 week from baseline (Screening).

To analyze the change in FVC on Week 24 ± 1 week versus baseline, a t-test or a Mann–Whitney test will be used, depending on the type of data distribution.

To analyze the number of chronic pulmonary disease exacerbations within 24 weeks ± 1 week of the therapy, Poisson regression is suggested. For each group of patients, the average number of exacerbations (M±SD) recorded for 24 weeks ± 1 week will be presented. Subject's gender, age, FEV_1 baseline (40–60% or more than 60% to 100% of the due value), presence of concomitant diseases, etc., can be used as covariates.

Additionally, the number and proportion of patients with one, two or three, or more exacerbations will be tabulated in total and by baseline FEV_1 stratification. The intergroup comparison of these data will be performed by the χ^2-criterion or Fisher's exact test (if the expected frequency in any cell is less than 5). The number of days to chronic pulmonary disease exacerbation will be analyzed using the Kaplan–Meier method, and also using the Cox regression model.

The intragroup analysis of body weight change at Week 24 ± 1 week compared to baseline will be performed using the paired t-test or Wilcoxon test (with significant deviations from a normal distribution). The intergroup comparison will be performed using the t-test or Mann–Whitney test.

The data on change of the average score of the subscales "Symptoms", "Activity", "Impacts", and the average total score of the St. George's Respiratory Questionnaire, version 2.2, at Week 24 ± 1 week compared to baseline will be analyzed using non-parametric methods: Wilcoxon test — for intragroup comparison and Mann–Whitney test — for intergroup comparison.

Analysis of PK parameters

Descriptive statistics will be provided for each PK parameter (C_{max}, T_{max}). A formal intergroup comparison, if necessary, will be carried out using a nonparametric Mann–Whitney test, since a significant deviation from the normal distribution is expected.

Safety analysis

The description of adverse events (AEs) will be presented according to the following scheme:

- Nature of the adverse event
- Severity of manifestation
- Duration
- Probable connection with the study substance
- Outcome

Adverse events will be coded in accordance with the Medical Dictionary for Regulatory Activities (MedDRA). The number, as well as the proportion of patients having developed a particular AE will be given for each AE/SAE. Intergroup comparison of categorical parameters (frequency of AE development, incidence of abnormalities based on results of physical examination, vital signs, laboratory and instrumental studies, patient diaries, and frequency of subjective complaints) will be carried out using the χ^2-criterion or Fisher's exact test (if the expected frequency in any cell will be less than 5). The number and proportion of patients with any abnormalities will be given for each parameter.

Assessment of the quantitative parameters (vital signs and laboratory parameters, ECG data) over time by Week 24 ± 1 week of treatment from baseline will be carried out using the Friedman test. If any statistically significant *post-hoc* differences are revealed, a pairwise comparison will be performed using the paired t-test or Wilcoxon test.

A generalized linear model (GLM) will be built to compare the dynamics of these parameters between the groups.

Data on the level of anti-drug antibodies against dornase alfa within 24 weeks ± 1 week of the therapy in patients of the active treatment group (Tigerase) and the reference group (Pulmozyme®) will be presented by means of descriptive statistics.

15.2 Subset Calculation

Within this phase II/III study, the Forced Expiratory Volume in 1 second (FEV_1) at Week 24 ± 1 week versus baseline was used as the primary efficacy endpoint. The efficacy of the investigational product Tigerase (GENERIUM JSC, Russia) will be compared with the efficacy of Pulmozyme® (F. Hoffmann-La Roche Ltd., Switzerland) in treatment of CF patients. However, since this disease is very rare, the number of patients available in Russia does not allow conducting a study using a generally accepted format of non-inferiority testing. In this regard, it is planned that the study will include all available patients aged 18 and older with an established diagnosis of cystic

fibrosis according to the relevant registries. According to preliminary information, up to 125 such patients can be available.

In this study, it is proposed to test the null hypothesis about the equality of efficacy of the two products, H_0: ΔFEV_1 (Tigerase) = ΔFEV_1 (Pulmozyme®), where ΔFEV_1 represents the change in Forced Expiratory Volume in 1 second (absolute %) on Week 24 ± 1 week compared to baseline in the Tigerase and Pulmozyme® groups, respectively.

The alternative hypothesis is two-sided, H_a: ΔFEV_1 (Tigerase) ≠ ΔFEV_1 (Pulmozyme®). The null hypothesis will be tested using a two-tailed test at a significance level of 0.05 ($Z_\alpha = 1.96$). The planned study power will be 80% ($Z_\beta = 0.84$), which will allow detecting a difference (δ) of 5% or more between the compared groups for ΔFEV_1, with the expected standard deviation of 10% for this parameter (σ) (Table 3):

$$\delta^2 = \sqrt{\frac{4(Z_\alpha + Z_\beta)^2 \sigma^2}{n}}$$

Table 3. Expected magnitude of differences for the primary efficacy endpoint (ΔFEV_1), which can be detected with the specified subset size

n	σ (%)	α; 1-β (%)	δ (%)
70	10	0.05; 80	6.7
80	10	0.05; 80	6.3
90	10	0.05; 80	5.9
100	10	0.05; 80	5.6

15.3 Deviation from the Statistical Data Analysis Plan

The statistical data analysis plan will be compiled and approved before the database lock. The statistical data analysis plan will contain a detailed description of cumulative data compilation and data analysis procedure (primary analysis and subanalysis), as well as clear scheduling of these kinds of analysis. All deviations from the initial statistical data analysis plan will be described and substantiated in the Clinical Study Report.

15.4 Adverse Events

The data on adverse events will be encoded using the current version of the MedDRA medical dictionary. The incidence of adverse events during treatment will be calculated for each organ system, according to the main diagnosis, treatment group, and in terms of the absolute and relative number of patients who developed adverse events. Summary data on the severity of adverse events and their relationship with the treatment will be presented for each organ system and by the principal diagnosis.

The cumulative data on the withdrawal of patients due to adverse events will be provided for each organ system and by the principal diagnosis.

The data on serious adverse events will be presented as lists and as cumulative data for each organ system and by the principal diagnosis.

16 SOURCE DATA, DATA HANDLING, MAINTENANCE, ARCHIVING, AND RECORD RETENTION

Sources and access to source data, data handling, documentation maintenance

As the subjects undergo scheduled visits and the investigators fill out the CRFs, the Sponsor's authorized monitor will verify the CRFs and the source documentation.

The study should be conducted in accordance with the Protocol and standard operating procedures of the Sponsor. If amendments to the Protocol are required, the Investigator and the Sponsor should agree on these amendments and document them as an amendment to the Protocol subject to the approval by the Ethics Committee.

All nvestigators are requiredi to fill out the basic documentation of the clinical study — the original medical records and the CRFs.

The Investigator is responsible for the complete and accurate filling of the CRFs. All data recorded in the CRFs should also be included in the original medical records of a subject in printed form or in the form of records made by the Investigator or another authorized person at the clinical center.

In accordance with the source documentation, all proposed data on the participation of a subject in the study should be recorded in the CRFs. The CRFs should also contain the data regarding the subject's completion of participation in the study. The CRFs shall be filled out not later than 5 days after the subject's visit to the study center.

All missing data in the source documentation and in the CRFs should be supported with an explanation in the form of comments. The CRFs shouldbe signed by the Principal Investigator. These signatures verify reliability of the information presented in the CRF.

The Investigator is obliged to provide direct access to the subject's data and study records when conducting study-related monitoring, audit, and inspection by the authorized regulatory bodies and to give all necessary explanations to the representatives of these organizations.

All information on the study and collected data are strictly confidential. The Investigator is only entitled to provide information on the study to persons not directly involved in it with the Sponsor's permission.

The Final Report consisting of a statistical and clinical report is formed after the database lock date and completion of the statistical processing of the study data. The Final Report shouldbe signed by the Principal Investigator of the clinical center, who confirms the results and conclusions of the study attaching it with this organization's seal.

16.1 Record Archiving and Retention

At the clinical center, all records and documents related to the clinical study and contained in the Investigator’s file, including informed consent forms, logs, subject registries, as well as the subject’s source medical records must be kept for 15 years after completion of the study. The study Sponsor provides retention of all materials of the clinical study throughout the entire life cycle of the investigational product.

Archival data can be stored as photocopies or on optical/electronic storage media. The Principal Investigator of the clinical center is obliged to immediately inform the Sponsor about the destruction or changes in the location of the archived materials of the clinical study.

17 ETHICAL ASPECTS OF THE STUDY

17.1 General Provisions

This study will be conducted in accordance withto the Declaration of Helsinki of the World Medical Association, Ethical Principles for Medical Research Involving Human Subjects, dated 1964, with subsequent amendments and additions; National Standard of the Russian Federation GOST R 52379-2005, Good Clinical Practice, dated September 25, 2005, Federal Law of the Russian Federation No. 61-FL, On Circulation of Medicines (with subsequent amendments and supplements), dated April 12, 2010, as well as guidelines of the International Conference on Harmonization — Good Clinical Practice (ICH GCP).

17.2 Procedure for Informed Consent Obtaining

Prior to enrollment into the study, the subject should be provided with a written or oral explanation of the purposes, objectives, and methods of the study, as well as expected benefit and potential risk associated with the participation in the study. In addition, the subjects should be informed about the voluntary nature of participation in the study and that the subject has a right to withdraw from the study at any time, and that such withdrawal will not affect the quality of the medical care provided to him/her. Although the subject is under no obligation to report the reasons for his/her withdrawal, the Investigator should try to find them out, however, without prejudice to the subject's rights. The subject's consent should be obtained prior to any study procedures.

Processing of the data collected during the study should be performed while maintaining the confidentiality of the subjects' data. The subjects should be informed about the purposes of the planned computer processing of the data and terms of these data publication (for example, for presentation at medical conferences, in journal articles, and other publicly available sources), presented only in the aggregated form that does not allow any identification of the subject.

Subjects should be informed that authorized representatives of healthcare authorities and the Sponsor will have access to their confidential medical information for monitoring, inspection, and audit. Subjects are guaranteed strict confidentiality of all information that allows identifying the subject, and non-disclosure of such information. The Informed Consent Form (Patient Information Leaflet) shall be filled out in duplicate, signed, and dated by the subject and the Investigator in their own hand. The Investigator should keep one copy of the signed Informed Consent Form in the Investigator's File, and the other copy is handed over to the subject.

17.3 Study Subjects Confidentiality and Identification

The confidentiality of identifiable records will be maintained in accordance with the right to privacy and confidentiality in accordance with regulatory requirements. Personally identifiable records will be protected and kept confidential and may only be disclosed to the extent it is allowed by the legislation. If the study results are published, the confidentiality of the information regarding the subjects' data will be preserved.

17.4 Recruitment of Subjects from Specific and Vulnerable Populations

The criteria of inclusion/exclusion stipulate participation in the study of the women with preserved childbearing potential if they provide consent for the use of reliable contraception methods specified in Section 4.2.

18 STUDY MONITORING AND STUDY QUALITY ASSURANCE

Regular visits of the study monitor before, during the study, and upon the study completion contribute to the success of the study and serve as a guarantee for collecting accurate data, timely detection of possible errors, documenting the clinical study process, and ensuring the protection of the rights of study subjects and compliance of the study with the requirements of international and Russian laws.

Routine monitoring of the study includes:

- Confirmation of proper study conduct and documenting the process of obtaining informed consent, as well as screening and enrollment of subjects in the study;
- Verification of the CRF data and source medical records of the study subjects;
- Confirmation of documenting and timely reporting of AE data during the study; confirmation of the clinical center personnel's compliance with the requirements for the implementation of diagnostic and therapeutic procedures of the study protocol;
- Confirmation of documenting the supply, storage, distribution, and destruction of the investigational product/study materials;
- Confirmation of the competence of the clinical center personnel and external laboratory personnel required for the study;
- Confirmation of the compliance of diagnostic and laboratory equipment with the requirements for their safe and adequate use during the study;
- Confirmation of the Investigator's cooperation with the local ethics committee on questions pertaining to the study safety and making amendments to the study protocol agreed by the Sponsor.

In accordance with legal requirements, the Sponsor or authorized state bodies have the right to inspect (audit) the study materials and equipment as well as the study records. The Investigator should provide access to the documentation and all the necessary information to those authorized to conduct an audit or inspection.

19 PROTOCOL AMENDMENT, DEVIATION, AND VIOLATION

Investigators’ signatures on the signature page of the protocol constitute written confirmation of agreement to conduct the study in accordance with this protocol. Amendments and supplements may be introduced into the study materials during the clinical study. Such changes and supplements should be considered as amendments.

Protocol amendment means a written description of changes or a formal explanation of the text of the clinical study protocol. Amendments can be significant and minor. Prior to entry into force, each protocol amendment should be approved in accordance with the established internal standard operating procedures of the Sponsor, after which it is approved by the Ministry of Health of the Russian Federation and local ethics committees and signed by the Investigator.

Order of the Ministry of Health of the Russian Federation No. 775 dated August 31, 2010, On Approval of the Procedure for Processing the Statement about the Need to amend the Clinical Study Protocol of the Medicinal Product, defines the list of significant/minor amendments and the procedure for the relevant materials submission for expert examination.

Amendments to clinical study materials should be considered significant if they may affect the purposes, forms of management, methodology of conduct, statistical methods of processing the clinical study results, and measures to ensure the safety of the patients participating in the study.

Amendments to clinical study materials should be considered minor if they do not affect the purposes, forms of management, methodology of conduct, statistical methods of processing the clinical study results, and measures to ensure the safety of the patients participating in the study.

If there is a need to amend this protocol, the Sponsor sends a notification of the need to amend the clinical study protocol to the Ministry of Health of the Russian Federation. The decision to make changes or refuse to make changes is made by the Ministry of Health of the Russian Federation after an examination of the updated materials submitted.

Protocol amendments should be stored along with the initial protocol version. The number of the amendment and its effective date should be indicated on the title page of the protocol. All deviations from the Protocol during the study should be documented during the monitoring of the clinical study.

20 STUDY FINANCIAL AND ADMINISTRATIVE CONTEXT

The Study Sponsor should ensure life and health insurance of the study subjects. In accordance with the applicable legislation, the subjects should be compensated for the damage in case of a threat to life and health associated with the use of the study/reference product.

22 APPENDIX 1. STUDY PROCEDURE SCHEDULE

		Treatment period								
**Visit**	**V1**	**V2**	**V3**	**V4 (TC*)**	**V5**	**V6 (TC*)**	**V7**	**V8 (TC*)**	**V9 EOT/ET****	**Unscheduled visit*****
**Day**	**Screening -21 to Day -1**	**Day 1**	**Day 7**	**Day 28**	**Day 56**	**Day 84**	**Day 112**	**Day 140**	**Day 168**	**–**
**Week**	**W-3**	**W0**	**W1**	**W4**	**W8**	**W12**	**W16**	**W20**	**W24**	**–**
**Visit window**	–	–	**±2 days**	**±3 days**	**±7 days**	**±7 days**	**±7 days**	**±7 days**	**±7 days**	–
Patient's informed consent	X									
Evaluation of compliance with inclusion/exclusion criteria	X									
Randomization		X								
Demographics, life history	X									
Medical history	X									
Height and body weight measurement[1]	X[1]	X	X		X		X		X	
Physical examination	X	X	X		X		X		X	
Evaluation of vital signs	X	X	X		X		X		X	
Sweat test[2]	X[2]									
DNA probe assay[3]	X[3]									
ECG	X								X	
Complete blood count	X				X				X	
Biochemistry	X				X				X	
Serology (HIV, Hepatitis B and C)[4]	X[4]									
Blood test for anti-drug antibodies (IgG, IgE), including neutralizing antibody activity	X				X				X	
Urinalysis	X								X	
Urine test for human chorionic gonadotropin with a test-strip	X				X				X	

		Treatment period								
**Visit**	**V1**	**V2**	**V3**	**V4 (TC*)**	**V5**	**V6 (TC*)**	**V7**	**V8 (TC*)**	**V9 EOT/ET****	**Unscheduled visit*****
**Day**	**Screening -21 to Day -1**	**Day 1**	**Day 7**	**Day 28**	**Day 56**	**Day 84**	**Day 112**	**Day 140**	**Day 168**	**–**
**Week**	**W-3**	**W0**	**W1**	**W4**	**W8**	**W12**	**W16**	**W20**	**W24**	–
**Visit window**	–	–	**±2 days**	**±3 days**	**±7 days**	**±7 days**	**±7 days**	**±7 days**	**±7 days**	–
(for women with preserved childbearing potential)										
Spirometry	X		X		X		X		X	
Sputum sampling to study pharmacokinetics of the study/reference product[5]		X[5]								
Assessment of clinical symptoms of chronic pulmonary disease exacerbation			X		X		X		X	
Interviewing the patient to assess clinical symptoms of chronic pulmonary disease exacerbation[6]				X[6]		X[6]		X[6]		
Assessment of the quality of life according to the St. George's Respiratory Questionnaire, version 2.2	X								X	
Product inhalation[7]		X[7]	X[7]	X[7]	X[7]	X[7]	X[7]	X[7]	X[7]	
Product dispensing		X	X		X		X			
Product return and accountability			X		X		X		X	
Handing over the patient diary		X	X		X		X			
Returning and checking the patient diary			X		X		X		X	
Registration of the concomitant therapy	X	X	X	X	X	X	X	X	X	
Assessment of exclusion criteria			X		X		X			
AE registration			X	X	X	X	X	X	X	

[1] The height is measured once only at the Screening visit.

[2] The sweat test is performed at the Screening visit in the absence of documented confirmation of cystic fibrosis diagnosis. The sweat test procedure is described in Section 10.10.

[3] DNA probe assay to detect mutation in the CFTR gene is performed at the Screening Visit in the absence of documented confirmation of cystic fibrosis diagnosis (Section 10.11).

[4] The results of serological tests (HIV, hepatitis B and C) received no later than 30 days prior to the Screening Visit are acceptable.

[5] Sputum samples to study pharmacokinetic parameters of the study/reference product will be collected from patients having provided a consent for participation in the additional part of the study 1 hour ± 15 min before the inhalation of the study/reference product (after the patient's randomization), then 15 min ± 5 min after the end of inhalation of the study/reference product and 1 hour 45 min ± 15 min after the end of inhalation of the study/reference product.

[6] The list of questions for assessment of clinical symptoms of chronic pulmonary disease exacerbation asked by the clinical investigator or an employee of the study center authorized by him/her is presented in Section 10.6.

[7] Patients shall perform inhalations of the study/reference product at home, except for the patients who provided consent for participation in the additional part of the study for assessment of the DNase concentration in the sputum. These patients will receive an inhalation of the study/reference product at Visit 2 (Day 1) in hospital settings.

*TC — telephone call.

**EOT/ET — end of therapy visit / early termination visit.

***During unscheduled visits, the required procedures, including laboratory and instrumental examinations, will be performed by the decision of the Investigator.

23 APPENDIX 2. QUESTIONNAIRE FOR PATIENTS

St. George's Respiratory Questionnaire, v. 2.2 (*)

Choose one answer for each statement:

1. **Over the past year, I have coughed:**
   Often
   Sometimes
   Rarely
   Once
   No
2. **Over the past year, I have brought up phlegm (sputum):**
   Often
   Sometimes
   Rarely
   Once
   No
3. **Over the past year, I have had shortness of breath:**
   Often
   Sometimes
   Rarely
   Once
   No
4. **Over the past year, I have had attacks of wheezing:**
   Often
   Sometimes
   Rarely
   Once
   No
5. **During the past year, how many severe or very bad unpleasant attacks of respiratory disease exacerbation have you had?**
   More than 3 attacks
   3 attacks
   2 attacks
   1 attack
   None
6. **How long did the worst attack last?**
   Go to question 7 if you had no severe attacks.
   A week or more
   3 or more days
   1–2 days
   Less than a day
7. **Over the past year, in an average week, how many days free of respiratory symptoms (with little manifestation of the respiratory disorder) have you had?**
   No good days
   1–2 good days
   3–4 good days

Almost all days were good
All days were good

8. **If you have expectoration, is it worse in the morning?**
No/Yes

9. **How would you describe your respiratory system condition?**
The most important problem I have
Causes me quite a lot of problems
Causes me a few problems
Causes no problems

10. **When you worked did your respiratory disorders make you leave your job (retire) early?**
My pulmonary problems made me leave my job
My pulmonary problems interfere with my work or made me change my job
My pulmonary problems do not affect my work

11. **Questions about what activities usually make you feel breathless these days**
Sitting or lying still
Getting washed or dressed
Walking around the home
Walking over the flat ground
Walking up a flight of stairs
Walking up hills
Playing sports or games

2. **Additional questions about your cough and breathlessness these days**
My cough hurts
My cough makes me tired
I get breathless when I talk
I get breathless when I bend over
My cough or breathing disturbs my sleep
I get exhausted easily

3. **Questions about other effects your respiratory disorder may have on you these days**
My cough or breathing is embarrassing in public
My respiratory disorder is a nuisance to my family, friends, or neighbors
I get afraid or panic when I cannot get my breath
I feel that I am not in control of my respiratory disorder
I do not expect my respiratory disorder to get any better
I have become frail or disabled because of my disease
Physical exercise is not safe for me
Everything seems too much of an effort

4. **Questions about your medication**

If you are receiving no treatment for respiratory disorders, go straight to question 15.

My medication does not help me very much
I get embarrassed using my medication or inhalers in public
My medication causes unpleasant side effects (additional health problems)
My treatment interferes with my life a lot

5. **Questions about how your breathing may affect your daily activities**

Put "yes" or "no" answer next to each statement.

I take a long time to get washed or dressed
I cannot take a bath or a shower, or I take a long time
I walk more slowly than other people or have to stop for rests from time to time

Work such as housework takes a long time, or I have to stop for rests
If I walk up one flight of stairs, I have to go slowly or stop
If I hurry or walk fast, I have to stop or slow down
My respiratory problems make it difficult for me to walk up hills, carry things upstairs, light gardening (for example, weeding), dance
My respiratory problems make it difficult for me to carry heavy loads, dig the garden or shovel snow, jog or walk fast (5 miles per hour), play tennis or swim
My respiratory problems make it difficult for me to do heavy manual work, run, cycle, swim fast, or play competitive sports

6. **We would like to know how your respiratory disorder usually affects your everyday life:**
Due to my respiratory disorder, I cannot play sports or active games
Due to my respiratory disorder, I cannot go out for entertainment or recreation
Due to my respiratory disorder, I cannot go out of the house to do the shopping
Due to my respiratory disorder, I cannot do housework
Due to my respiratory disorder, I cannot move far from my bed or chair
7. **Choose the sentence that best describes the impact of lung disease on your life.**
It does not stop me doing anything I would like to do
It stops me doing one or two things I would like to do
It stops me doing most of the things I would like to do
It stops me doing everything I would like to do

Scoring algorithm

Total
Three component scores are calculated: symptoms, activity, impact. One Total score can be calculated.

Principle of calculation

Each Questionnaire response has a unique empirically derived value. The minimum possible value is zero, and the highest is 100.

Each component of the questionnaire is scored separately in three steps.

1. Sum up the values for all items with positive responses.
2. The values of missed items are deducted from the maximum possible value of each component. The values of all missed items are deducted from the maximum possible value of the Total score.
3. The score is calculated by dividing the summed values by the maximum possible weight for this component and expressing the result as a percentage:

$$\text{Score} = \frac{\text{Summed values of positive items in this component}}{\text{Sum of values of all items in this component}} \times 100$$

The Total score is calculated in a similar way:

$$\text{Score} = \frac{\text{Summed values of positive items in the Questionnaire}}{\text{Sum of values of all items in the Questionnaire}} \times 100$$

Sum of maximum possible values for each component and Total:

- Symptoms 662.5
- Activity 1,209.1
- Impact 2,117.8
- Total 3,989.4

(Note: The given values are the maximum possible values that could be obtained for the worst possible state of the patient.)

It should be noted that the Questionnaire requests a single response to questions 1–7, 9–10, and 17. If multiple responses are given to one of these questions, then calculate the average value acceptable for the positive responses to that question. We believe it is a better approach than losing the obtained data. This calculation technique was proposed and verified in our studies. Clearly, it is better to prevent such multiple responses from occurring, but it is difficult to completely prevent such occurrence. This method is used in the Excel calculator.

Symptoms component

It is calculated from the summed values of positive responses to questions 1–8.

Activity component

It is calculated from the summed values of positive responses to questions 11 and 15.

Impact component

It is calculated from the summed values of positive responses to questions 9–10, 12–14, and 16–17.

TOTAL score

The Total score is calculated by summing all positive responses in the Questionnaire and expressing the result as a percentage of the total value of the Questionnaire (as shown above).

Handling missed items

It is better not to miss items, and any missing items are the fault of the experimenter, not the patient. We have examined the effect of missing items and recommend the following methods:

Symptoms

The Symptoms component will tolerate a maximum of 2 missed items. The value of the missed item is subtracted from the total possible value of the Symptoms component (662.5) and from the Total value (3,989.4).

Activity

The Activity component will tolerate a maximum of 4 missed items. The values of the missed items are subtracted from the total possible value of the Activity component (1,209.1) and from the Total value (3,989.4).

Impact

The Impact component will tolerate a maximum of 6 missed items. The values of the missed items are subtracted from the total possible value of the Impact component (2,117.8) and from the Total value (3,989.4).

***Jones PW, Quirk FH, Baveystock CM. The St George's Respiratory Questionnaire. Respir Med. 1991; 85 Suppl B: 25–31.**
